# Assembling the climate story: use of storyline approaches in climate‐related science

**DOI:** 10.1002/gch2.202200183

**Published:** 2023-05-23

**Authors:** Eulàlia Baulenas, Gerrit Versteeg, Marta Terrado, Julia Mindlin, Dragana Bojovic

**Affiliations:** ^1^ Barcelona Supercomputing Centre (CNS‐BSC) Plaça d'Eusebi Güell, 1‐3 Barcelona Barcelona 08034 Spain; ^2^ Departamento de Ciencias de la Atmósfera y los Océanos Facultad de Ciencias Exactas y Naturales Universidad de Buenos Aires Buenos Aires Argentina; ^3^ Centro de Investigaciones del Mar y la Atmósfera Consejo Nacional de Investigaciones Científicas y Técnicas Universidad Nacional de Buenos Aires Buenos Aires Argentina; ^4^ Instituto Franco Argentino sobre estudios de Clima y sus impactos (IFAECI‐UMI3351) Centre National de la Recherche Scientifique Buenos Aires Argentina

**Keywords:** climate storylines, constructivism, scenario‐based approaches, scenarios, transdisciplinarity

## Abstract

Storylines are introduced in climate science to provide unity of discourse, integrate the physical and socioeconomic components of phenomena, and make climate evolution more tangible. The use of this concept by multiple scholar communities and the novelty of some of its applications renders the concept ambiguous nonetheless, because the term hides behind a wide range of purposes, understandings, and methodologies. This semi‐systematic literature review identifies three approaches that use storylines as a keystone concept: scenarios—familiar for their use in IPCC reports—discourse‐analytical approaches, and physical climate storylines. After screening peer‐reviewed articles that mention climate and storylines, 270 articles are selected, with 158, 55, and 57 in each category. The results indicate that each scholarly community works with a finite and different set of methods and diverging understandings. Moreover, these approaches have received criticism in their assembly of storylines: either for lacking explicitness or for the homogeneity of expertise involved. This article proposes that cross‐pollination among the approaches can improve the usefulness and usability of climate‐related storylines. Among good practices are the involvement of a broader range of scientific disciplines and expertise, use of mixed‐methods, assessment of storylines against a wider set of quality criteria, and targeted stakeholder participation in key stages of the process.

## Graphical abstract

This article presents a semi‐systematic literature review that identifies three storyline‐reliant approaches in climate‐related science. The review highlights how the divergent approaches have a wide range of methodologies to assemble storylines, but currently exist independently of each other. The article discusses ways in which cross‐pollination based on good practices could benefit each.

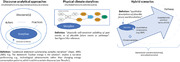

## 1 Introduction

A warming climate puts strenuous demands on societal actors from all sectors and scales, who are requested to rapidly devise adequate responses to ensure a livable future for climate‐impacted communities. To support such an endeavor, one of the aims of the climate science produced nowadays is to improve projections and predictions about the expected extent, location, direction, and timing of climatic changes.^[^ ^1^, ^2^ ^]^ The evolution of our climate will depend on fundamental changes in social systems, which are determined by decisions on various sectors such as energy or food. Accordingly, several approaches have emerged in climate‐related science with the aim to envisage future trends whilst accounting for a reacting society. The increasing use of such approaches has been spurred additionally by two parallel paradigm changes: the recognition that both the scientific method and the communication of science rely on narrative processes and the importance of transdisciplinarity for producing *usable* climate science.^[^ ^3^, ^4^, ^5^, ^6^, ^7^, ^8^ ^]^

Scholars have nonetheless identified a usability gap between climate science and climate action, understood as the “differences that might exist between what scientists might think is useful, and what is actually usable in practice.”^[^ ^9^ ^]^ This gap might stem from differences in types of knowledge, resources, and capacities between scientists and climate information users, the discrepancy between available and needed temporal and spatial scales, and the lack of real interdisciplinarity.^[^ ^10^, ^11^, ^12^, ^13^, ^14^, ^15^, ^16^ ^]^ The concept of “storylines” is one vehicle that scholars can use when aiming to address this gap, as it allows us to take into consideration and operationalize the social dimension of climate change. Especially, the use of storylines may support decision‐making by better dealing with tangible problems, uncertain facts, disputed values, and the urgency of action, as well as exploring new ways of science communication.^[^ ^4^, ^17^, ^18^ ^]^

The latest recommendations on how to improve storyline‐reliant approaches stress the need to better connect across research communities and with users.^[^ ^19^, ^20^, ^21^ ^]^ Yet, the concept of “storylines” is far from being a unified concept, hampering the efforts to make it a useful tool for decision‐making. In this article, we conducted a semi‐systematic literature review that reveals the presence of three different approaches in climate‐related science, all using “storylines” but conceiving and operationalizing them differently: i) scenarios (or the story‐and‐simulation approach), ii) discourse‐analytical approaches, and iii) physical climate storylines. These approaches use similar terminology but consist of different objects, including the concepts of narratives, frames, or scenarios. Due to upward publication trends in recent years in the usage of “storylines” and the calls for improving interdisciplinarity in climate‐related science, it is important to open the black box and disclose these differences to research communities, supporting the exchange of good practices.^[^ ^22^ ^]^ Even though there have been several reviews on scenario‐based methodologies, to our knowledge none has taken a comparative approach nor addressed storylines specifically, showing a gap in the literature. This is the case regarding the reviews that cover scenario‐related methodologies such as impact assessments,^[^ ^23^, ^24^, ^25^ ^]^ as well as for discourse‐analytical approaches.^[^ ^26^ ^]^

**Figure 1:**
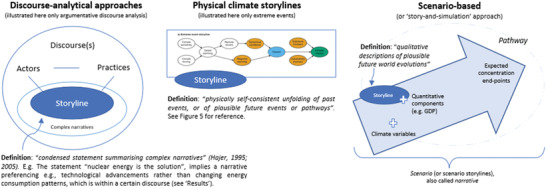
Three different forms to conceptualize “storyline” in climate‐related literature.

Differences can already be distinguished from the definition each of these approaches has of “storyline.” Starting with scenario‐based approaches, the IPCC AR5 report^[^ ^27^ ^]^ defined *storylines* as “qualitative descriptions of plausible future world evolutions, describing the characteristics, general logic and developments underlying a particular quantitative set of scenarios”. *Scenario storylines* are further defined as “a narrative description of a scenario (or family of scenarios, such as the SSPs), highlighting the main scenario characteristics, relationships between key driving forces and the dynamics of their evolution”.^[^ ^28^ ^]^ The recent IPCC AR6 introduced the definition of “Physical climate storylines,” based on Shepherd and colleagues^[^ ^18^ ^]^ and understood as a “physically self‐consistent unfolding of past events, or of plausible future events or pathways”. Although the IPCC AR6 WG1 report underlines the interchangeable use of storylines and narratives, it follows the PCS definition of storylines through multiple chapters of its assessment report.^[^ ^29^ ^]^ In scenarios and physical climate storylines, the terms narratives, scenarios, and storylines are often used interchangeably^1^, although as we detail in the Results section, they consist of different objects and components.^[^ ^18^, ^30^, ^31^, ^32^ ^]^ Discourse‐analytical approaches, primarily in the field of argumentative policy analysis, also use the concept of storyline, but differently than the other two approaches. Hajer^[^ ^33^ ^]^ defined the concept as “a condensed form of narrative in which metaphors are used”. This definition stems from the observation that actors might use wording implying certain meanings, and under common storylines, discourse coalitions might be formed with preferences and strategies for a specific course of action.^[^ ^33^, ^34^ ^]^ **Figure** **1** gives a visual representation of the storyline definitions in the approaches.

Accordingly, we seek to answer two main research questions. First, how do different scholar communities treat and produce storylines in connection to climate‐related phenomena? And second, how can cross‐pollination among these communities enable the assembly of more useful and usable climate‐related storylines? The benefits of a better understanding of what storylines are and how they are produced can support greater collaboration across scholar communities, as well as encourage scientists to go a step further and embrace other types of knowledge and methodologies to conduct its assembly.

In the next section, we detail how we conducted the semi‐systematic literature review. The results section provides an overview of the general trends in the identified literature and dives into each approach. Finally, the discussion introduces the ways in which cross‐pollination among the approaches could help address some of the weaknesses identified within each, which stop them short of being a useful tool for climate‐related decision‐making of a broader set of societal actors.

## 2 Methodology

In this article, we review climate‐related literature that used “storylines” in their theoretical framework, conceptualization, or operationalization. The study aims to address the heterogeneity of applications existing in the literature, which might result in confusion and lack of awareness across disciplines of the presence of alternatives to their modus operandi. A literature review is recommended when studying and synthesizing existing concepts across disciplines.^[^ ^35^ ^]^ Semi‐systematic literature reviews are suggested especially when the topics discussed are studied and conceptualized differently among multiple disciplines.^[^ ^36^ ^]^

**Figure 2:**
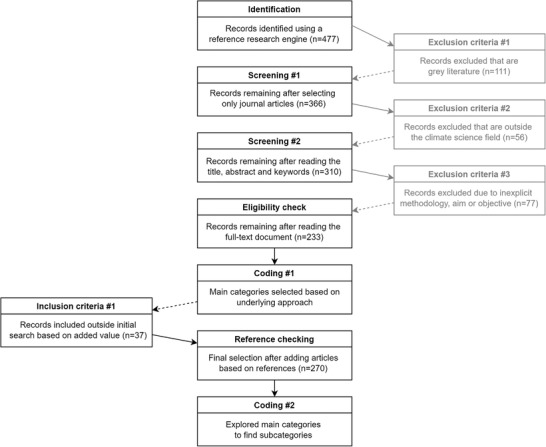
Methodological structure of the literature review.

The methodology structure described in this section follows an adapted version of the PRISMA protocol.^[^ ^37^ ^]^ The protocol stands for the Preferred Reporting Items for Systematic reviews and Meta‐Analyses. It followed a four‐phase flow diagram containing actions of identification, screening, eligibility, and inclusion, followed by our approach to data analysis and coding (**Figure** **2**).

### 2.1 Data Collection and Screening

The protocol started by identifying articles and screening them accordingly. The search string: (“climate”) AND (“storylines”) was used in the Scopus database using the Mendeley research engine and literature manager and it was contrasted with Google scholar, two databases often used in literature reviews.^[^ ^38^ ^]^ For the initial selection, peer‐reviewed articles written in English and published from January 2000 to June 2022 were included, which yielded 477 articles (Figure 2). Despite grey literature can be of high value in climate science as non‐academic disciplines are involved in its creation, the analysis was focused on peer‐reviewed literature due to its completeness of identifiers that allows for systematic retrieval, reproduction, and citation. Moreover, peer‐reviewed articles were relevant for the research question of identifying scholar communities based on storyline methodologies. Moreover, no standardized content quality was applied to grey literature and its heterogeneity might pose issues for its identification and processing^[^ ^39^, ^40^ ^]^

For the next screening step, the definition of “climate” from the IPCC Glossary was used, according to which, “Climate in a narrow sense is usually defined as the average weather (…). Climate in a wider sense is the state, including a statistical description, of the climate system”. Hence, articles that did not specifically address climate following the definition were excluded, despite containing the keywords in their corpus, such as articles related to education or cinema or even climate services.^[^ ^41^ ^]^ The authors used this criterion to assess the eligibility of the articles, which resulted in 310 relevant articles. Finally, the preselected articles underwent an eligibility check, after which the first coding started. During the eligibility check, a full‐text article screening was necessary if the abstract and keywords did not match the screening criterion, or if the methodology used or the article's aims and objectives were unclear. The combination of abstract and full‐text screening further narrowed the selection, resulting in 233 articles (Figure 2).

### 2.2 Data Analysis and Coding

The dataset of 233 articles was subject to the first coding round. Coding started by identifying recurring themes from delineated concepts in the abstracts. Codes were created from keywords found in the abstract and grouped into categories, analyzing the content of each article. MaxQDA was used, a qualitative data (nonnumerical, unstructured) analysis software.^[^ ^42^ ^]^ The coding served several purposes: 1) identifying general characteristics (**Table** **1**); 2) gathering similarities in abstracts (**Table** **2** and in the Supporting Information); and 3) assigning main categories from the literature sample.

**Table 1:** Coded keywords in order of frequency of appearance

Characteristic	Keywords
Sector	Multi‐sectoral; land use/vegetation; energy/emissions; water; climatology/atmospheric circulation; food systems/agriculture; risk management/planning; policy; biodiversity/ecosystems; media; GDP/economy/finance; nature and wildlife; history; health; entertainment; tourism; urban planning; climate services; geoengineering
Method	Model simulation; pathway transitions; presentation of concept/framework/toolbox; literature review; comparison study; discourse analysis; interviews/survey; participatory development; scenario‐based development; expert judgement; scenario workshops; network building; scenario discovery/optimization algorithms/analysis; regression‐based analysis; storytelling sessions; modelling chains; valuation; ethnographic work
Climate‐related field	Impacts; hazard; climate change mitigation; vulnerability; climate change adaptation; climate change drivers/pathways/theory; risk; exposure; risk (non‐IPCC definition)

**Table 2:** Storyline‐based approaches and coded keywords in order of frequency of appearance

Approach	Keywords
Scenario‐based	impact assessment; emission; scenario; development; SRES; socioeconomic; qualitative; planning; quantitative; pathway; global; vulnerability
Discourse‐analytical	adaptation; resilience; community; narrative; media; governance; policy; discursive; discourse; social; local; frame;
Physical climate storylines	physical; storyline; event; plausible; extreme; drivers; attribution; weather; atmospheric; circulation; uncertainty; conditional

For the general characteristics, the paper's sectoral application, methods used, and climate‐related field (based on the IPCC glossary), and approach‐specific keywords were searched, and if the production of storylines included participatory processes and what type, including methods used for this process (Table 1). Participatory processes can have different intensity degrees and thus may involve coproduction as a more intense approach. By coproduction, the definition of Bojovic and colleagues^[^ ^3^ ^]^ was followed by which it was considered “an iterative, interactive and collaborative process that brings together a plurality of knowledge sources to mutually define problems and develop usable products to address these problems”. The “product” in this article refers to the storyline, and coproduction included the storyline and the process of constructing it. For example, processes such as co‐exploration or co‐evaluation with a selected group of stakeholders can be included.

To identify the main categories of the approaches that use storylines in climate‐related science, studies were grouped by general methodology. These were contrasted with the literature and the labels were allocated from studies that defined them, coming up with the three main categories: discourse‐analytical approaches (DAA);^[^ ^26^ ^]^ physical climate storylines (PCS)—a term derived from the storyline approach in Shepherd et al.;^[^ ^43^ ^]^ and scenario‐based approaches (SB)—related to the initial emission scenarios, as well as identified generally as the “story‐and‐simulation” approach^[^ ^20^ ^]^ (Table 2).

Due to the recency of the PCS approach and the low initial number of obtained articles (23), a reference search outside the initial record aws conducted to find additional peer‐reviewed publications. With this step, the selection was expanded by 34 articles that complied with the mentioned criterion. The other two approaches, DAA and SB, had three articles published after the initial selection was completed. Including these recent articles and the additional PCS articles resulted in a total of 270 articles.

The coding technique also helped identify the subcategories of the three main approaches. It showed that DAA included both argumentative discourse analysis and frame analysis.^[^ ^26^ ^]^ PCS subcategories were identified based on the IPCC's sixth assessment report,^[^ ^44^ ^]^ except for a third recent addition to the subset of PCS studies, which was labeled "equilibrium climate sensitivity".^[^ ^45^, ^46^ ^]^ Finally, SB were subdivided based on their assembly of storylines. Despite departing from similar methodologies, the subcategories included different methods: downscaling and sectoral applications with SSP/RCP‐based storylines; non‐SSP‐based storylines, modeling and simulation studies; and mixed‐methods with co‐production of storylines.

To further support how the literature and retraced impactful articles were categorized, backward citation analysis was carried out to determine which articles frequently occurred in the reference lists of our final selection. From these articles, the five most cited were selected to give an insight into the reasoning for the three main categories (see Table S1, Supporting Information). The backwards citation showed that most shared SB references included terms like “scenario”, “narratives”, and “pathways”. PCS references used “storylines” and “tales”, while for DAA, the “discourse” term was dominant. These articles helped validate the labels and set the stage for their distinctive methodologies. We also include a table in the Supporting Information with the different definitions of these terms identified in each approach.

Finally, a bibliographic coupling map was created to visualize how each article related to another based on the number of references they share, similar to backward citation analysis. Depending on the link strength between groups of articles, clusters of associated articles are formed that can be compared to our categorization of storyline approaches in climate science (Figure 4). For this, the trimmed final selection of 265 articles with a valid publication identifier were imported into Lens, the online patent and scholarly literature search facility (https://www.lens.org/), to identify all the Lens IDs and acquire the references of each article. The data were exported in CSV format and uploaded to VOSviewer, a bibliographical mapping tool. A map was created based on bibliographical data, selected the bibliographic coupling of documents, and chose the full counting method. Before plotting the map, only connected articles were displayed and unconnected ones were discarded. Therefore, only 247 of the original 270 articles were shown, partly explained by the articles lacking identifiers and shared references. Although this mapping tool was quite informative for indicating related articles and the academic structure guided the categorization, it was not blindly followed to determine which article falls in which category. Instead, the map was combined with the backward citation analysis, backed up with definitions and coded keywords, as well as the authors’ judgement, to build a solid case for the main three categories of the approaches that use storylines.

## 3 Results

### 3.1 General Overview

#### 3.1.1 Publication Trends

**Figure 3:**
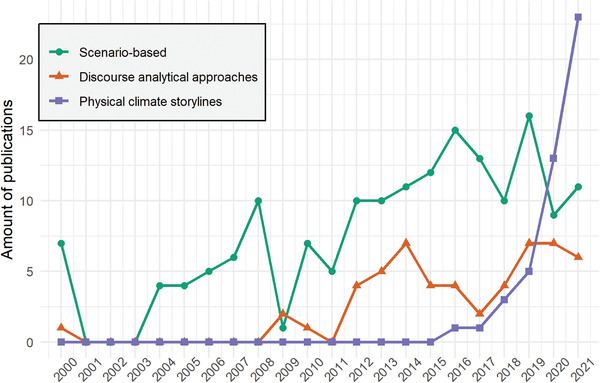
Publication trend of main storyline categories for 2000–2021.

**Figure** **3** shows the growth in the number of climate‐related peer‐reviewed articles utilizing the concept of storyline in the last few years. By "utilizing," it is meant that scholars operationalize or use the concept of “storyline” as part of a theoretical or empirical framework. As discussed in the previous section, we detected three main approaches that have these patterns: the literature on scenario‐based approaches (SB), discourse‐analytical approaches (DAA), and physical climate storylines (PCS). The categories contain 158, 55, and 57 journal articles, respectively. When plotting the published number of articles over time, it is obvious that the PCS approach is the most recent addition to the climate science domain. Although PCS contributes only to a minor portion of articles that utilize the term “storylines” in climate research, it has seen a steep increase in publications every year since the seminal Shepherd^[^ ^43^ ^]^ publication.

#### 3.1.2 Bibliographic Coupling Map

**Figure 4:**
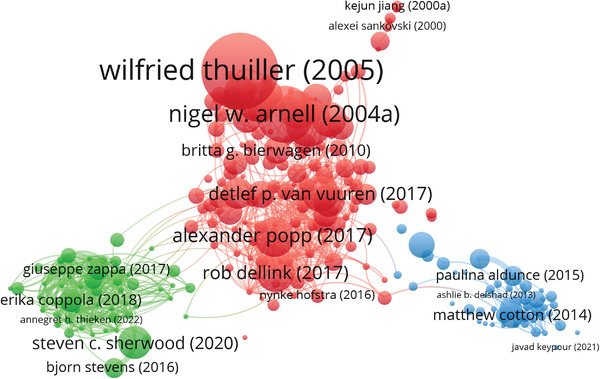
Bibliographic coupling map of the literature. The colors represent the approaches (red for SB, green for PCS, and blue for DAA). The size of the circles indicates the number of citations. Names displayed are referring to the first author.

**Figure** **4** below shows a bibliographic coupling map, showing the main categories found in the article. Bibliographic coupling is used to view the relatedness between articles by determining the number of shared references.^[^ ^47^ ^]^ The figure shows three distinct clusters of literature, similar to the three main storyline approaches. Although this map does not show the direct impact of specific influential articles on their approach, articles that have been cited more often (larger circle and font size) could have a larger reach and might be more impactful. Scenario‐based approaches are the oldest and most cited category in our sample. For PCS, Sherwood and colleagues^[^ ^45^ ^]^ was an important article for the IPCC AR6 report, Chapter 7, as the use of their proposed methodology allowed to narrow down uncertainty.^[^ ^48^ ^]^ Similarly, the DAA article by Cotton and colleagues^[^ ^49^ ^]^ was relevant in the energy discourse scholarly debate in the UK.

#### 3.1.3 Type of Journals

The type of journals that publish articles in relation to climate and using storylines from our final database of peer‐reviewed journal articles can be an indicator of the type of disciplines and scholarly communities working with each approach. Journals with tendencies towards physical sciences tend to publish PCS or SB, with the majority of articles in these categories published in *Science of the Total Environment* (12), *Technological Forecasting and Social Change* (9), *Environmental Research Letters* (7), *Earth System Dynamics* (6), and *Global Change Biology* (6). From those centered rather on social science, *Global Environmental Change* (14), *Environment and Planning C: Government and Policy* (4), and the more transdisciplinary journal *Climatic Change* (11), which has published articles from the three approaches, are determined. Out of the 10 journals that have the most publications on climate‐related storylines, four contained articles with at least two of the three main approaches. The other six journals published only articles of the same approach (**Table** **3**).

**Table 3:** Publications by journal and approach (for the 10 most common journals)

Journal	CA	SB	PCS
Global Environmental Change (14)	3	11	0
Science of the Total Environment (12)	0	12	0
Climatic Change (11)	1	8	2
Technological Forecasting and Social Change (9)	0	9	0
Environmental Research Letters (7)	0	3	4
Earth System Dynamics (6)	0	2	4
Global Change Biology (6)	0	6	0
Journal of Climate (6)	0	0	6
Weather & Climate Extremes (4)	0	0	4
Environment and Planning C: Government and Policy (4)	4	0	0
**Total**	**8/55**	**51/158**	**20/57**

Next, we describe the following details for each of the approaches and identified subcategories: the a) concept of “storyline”; b) main characteristics (i.e., what makes an article belong to the approach); c) aim for which it is used; d) how it is produced (methods); e) applications (e.g., sectoral, etc.). We detail this while paying attention to the approaches’ evolution and consistency over the years.

### 3.2 Description of Approaches

#### 3.2.1 Summary of Discourse‐Analytical Approaches

The studies found under this approach differ greatly from SB and PCS. In DAA, research objects may be shared meaning among actors, associated practices, and the underlying discourses in relation to climate change, science, and policy.^[^ ^26^, ^33^, ^50^ ^]^ Whilst discourse‐analytical studies from this perspective are not a unified field, many authors would agree that “knowledge is situated and that there are only partial truths”.^[^ ^4^ ^]^ Many of these studies, especially in the case of discourse analysis, depart from a social constructivist or critical realist ontology, which is the reason why the understanding, operationalization, and use of storylines changes greatly. In other words, whilst the other two approaches focus on and depart from physical climate science as the main established and valid knowledge or "proxies" for social constructs, discourse analysis focuses on the human and social understandings of such phenomena ‐including climate scientists as relevant actors with their accompanying discourses.

There are several discourse‐analytical approaches and from those, our review identified two which utilize the concept of “storyline”: Argumentative Discourse Analysis and Frame Analysis (for more on different discourse approaches^[^ ^26^, ^51^ ^]^):

##### Argumentative Discourse Analysis (ADA)

In total, 36 of 55 (65,5%) articles in this dataset used argumentative discourse analysis (see Supporting Information Table S1). The reason is that “storyline” as such is operationalized under the theoretical framework developed by Hajer,^[^ ^52^ ^]^ which sees storylines as a narrower, more concrete object than narratives. The same framework presents discourses as meaning patterns that enable or constrain certain courses of action because they mediate “how political entities and societies understand and act on certain social or physical phenomena that are negotiated in environmental policy making”.^[^ ^26^ ^]^

The following paragraph from Bastakoti and Davisen^[^ ^53^ ^]^ encompasses well the aims behind ADA:

“Analyzing storylines helps to identify and analyze how actors interpret, and utilize, a certain discourse. Hajer argues that shifts in storylines often drive policy development, making them an important factor in explaining policy change.^[^ ^28^, ^44^ ^]^ Storylines are not confined to any particular actor but are shared by the actors involved at different levels. (..). Once the storylines gain wider acceptance, they may help to unify actors and get closer to shared problem resolution approaches.^[^ ^28^ ^]^ Storylines serve as building blocks of discourses, however, not all discourses are equally influential to shape the debate. Power struggles between these discourses and coalitions of actors with their storylines shape our understanding and perceptions as discourses ultimately become institutionalised into policy.” (p. 4).

A group of storylines may form a narrative, which is described as a story ascribing meaning to social or physical phenomena by connecting a sequence of events and actions in a plot, including, excluding, and emphasizing problems, actors, and events and, thus providing an interpretation of who or what is significant Feldman 2004,^[^ ^54^ ^]^ p. 1047]. Thus, narrative is a more complex form than storyline, and both are under overarching discourses. While still being different, it is in the concept of narrative due to its temporal component, where this category is closer to SB than to PCS, especially in the studies that include participatory methodologies and actively distinguish narratives from storyline scenarios (see below, “Scenario‐based approaches”).

In methodological terms, Hajer,^[^ ^33^ ^]^ described the steps to conduct such an analysis, and these include: desk research, helicopter interviews, document analysis, interviews with key players, tracking in sites of argumentation (e.g., during Conference of the parties, “COPs”), analysis of positioning effects, identification of key incidents, analyses of practices in particular cases of argumentation, interpretation and a second visit to actors to corroborate with them the discourses they have been associated with.

Rosenbloom,^[^ ^55^ ^]^ building upon this and other discourse‐analytical approaches such as FA (below), is one of the few studies that further developed the concept of storylines to introduce indicators for their strength, being these “the believability of claims, the centrality of the issue, and the credibility of the messenger(s).” (p. 7). Similarly, Mayrhofer et al.^[^ ^56^ ^]^ used discourse *institutionalism* to merge the work of Hajer with that of Schmidt^[^ ^57^ ^]^ to further unravel how discourse coalitions work.

##### Frame Analysis

Frame analysis (FA) is another theoretical approach and method which includes “storyline” in their toolkit and understanding of framing. In these studies, framing means a central organizing idea or *story line* that provides meaning to an unfolding strip of events, weaving a connection among them. The frame suggests what the controversy is about, “the essence of the issue” [c.f. Gamson and Modigliani 1987:143, in 57 italics added]. The analysis of language and statements will thus also be necessary for such studies, equally as in ADA. The list of studies using FA in connection to climate is comparatively smaller though, comprising five articles.^[^ ^58^, ^59^, ^60^, ^61^ ^]^ Generally, the majority of studies use a similar set of techniques to identify storylines, including content and media analyses, interviews and surveys as well as participatory observation.

The few studies that diverged from the use of ADA or FA either did not provide any definition of storyline,^[^ ^62^, ^63^, ^64^, ^65^ ^]^ were not aligned with the meaning of DAA or FA, but shared a constructivist ontology^[^ ^66^, ^67^ ^]^ or used ADA without explicit mention.^[^ ^68^ ^]^ Some authors proposed alternative theoretical perspectives, such as in the case of Keypour et al.,^[^ ^69^ ^]^ who used strategic narrative theory (“a means by which political actors attempt to construct a shared meaning of the past, present, and future of international politics to shape the behavior of domestic and international actors”.^[^ ^70^ ^]^) from the field of international relations to study EU energy security policy.

In general, the main bulk of methodologies under this approach is qualitative, whilst examples of mixed‐methods or quantitative research designs are rare. For instance Campbell et al.^[^ ^63^ ^]^ used quantitative survey data combined with focus group discussions, and Eck & Feindt^[^ ^51^ ^]^ applied a quantitative approach to analyze the arguments forming ideological polarization between climate sceptics’ and climate activists’ blogs of two Conference of the Parties (COP15 and COP21).

#### 3.2.2 Summary of the Scenario‐Based Literature

Scenario methodologies are tracked back to the military and policy planning literature and relate to the efforts of studying different ways the future might unfold.^[^ ^71^ ^]^ These methodologies entered environmental and climate research over four decades ago, offering a way to combine social elements with ecological or climate‐relevant variables. The utilization of scenarios in IPCC Assessment Reports is possibly what makes this methodology the most numerous in our review of the use of storylines in connection to climate science, due to the efforts to be a tool that allowed for compatibility and comparability across models and assessments. There have been several changes in the IPCC's use of methodologies to construct scenarios.^[^ ^72^ ^]^ The Shared Socioeconomic Pathways (SSP)/Representative Concentration Pathway (RCP) framework constructs the RCPs parallel to that of SSPs, and integration at a later stage allows to bring forward impact, adaptation, vulnerability, and mitigation analyses.

Based on the extension of the SSPs or the use of a similar methodology, the literature on scenarios can be divided into three subcategories: the subset of articles that extends SSPs storylines, the subset that uses the concept of storylines consistent with the IPCC definition but builds their own qualitative descriptions as departure points and uses modeling and simulation; and the subgroup which also builds their own storylines but with participatory processes. Across all these articles, there is consistency in what “storyline” is, which respects the definition found in the IPCC, of “qualitative descriptions of plausible future world evolutions”, as referenced above. For simplification, “storyline” is a component of the SSPs, which adds in quantified social components (e.g., GDP), and gets integrated with RCPs: combined with the “stories” the pathways of each, are scenario storylines. The definitions of the terms and their use is the most consistent in this set of literature. Moreover, this literature uses storylines and narratives interchangeably,^[^ ^73^ ^]^ see also WGI AR6 Cross Chapter Box 6 in Chapter 1 and Sections 1.4.4 and 10.5.3 75], hence it can be the case that when mentioning “storylines” authors refer to the complete scenario storyline.^[^ ^74^, ^75^, ^76^, ^77^, ^78^, ^79^ ^]^ Similarly, these articles show consistency in the purpose of using storylines, which is a way to track possible trajectories, integrating socio‐economic concerns, and to support decision‐making.

##### SSP/RCP‐Based Storylines

For the first subset of articles (*N* = 95), the studies either downscale it, and/or apply it to specific sectors, or are methodological articles, as also observed in recent reviews on climate change scenarios.^[^ ^21^, ^80^ ^]^ Scales addressed are subnational;^[^ ^81^, ^82^, ^83^, ^84^, ^85^, ^86^, ^87^ ^]^ country‐resolved and comparative^[^ ^88^ ^]^ or national,^[^ ^89^ ^]^ regional,^[^ ^90^ ^]^ and multiscale.^[^ ^91^, ^92^ ^]^ Other applications within this subset are sectoral, with the main bulk adapting storylines to the water or agricultural sectors (Table B1shows these articles grouped by sectors). Generally, the studies use expert elicitation or judgment to further develop the storylines. In this subgroup (95), only seven studies have a mixed‐methods approach with a participatory approach to (re)build storylines.^[^ ^84^, ^90^, ^91^ ^]^ From this, some defend more intense participatory processes such as coproduction. For instance, Mitter et al.,^[^ ^19^ ^]^ proposed an iterative participatory approach to storylines assembly that enables storylines to meet quality criteria. These criteria include being plausible, consistent, salient, legitimate, rich, and creative. To achieve this, the process design requirements are those of being science‐driven, iterative, top‐down or nested, consecutive, participatory, and interdisciplinary.^[^ ^19^ ^]^ The articles that are methodological are either reflecting on ways to build SSPs,^[^ ^93^, ^94^, ^95^ ^]^ propose scenarios evaluation,^[^ ^96^, ^97^ ^]^ or add probabilistic projections.^[^ ^98^, ^99^ ^]^

##### Non‐SSP/RCP‐Based Storylines (Modeling and Simulation Studies)

In the second subset of non‐SSPs storylines, 29 articles (*N* = 29), there are studies that do not apply a participatory approach to the production of storylines. The majority of them often mention the criteria of "plausibility" and use the same methods to construct storylines, to afterward proceed with modeling or simulation techniques (Table B2 in Supporting Information).

##### Mixed‐Methods with Co‐Production

In this third subcategory, diverse mixed‐methods to construct storylines with participatory processes are used to build storylines: 34 studies, with 17 using mixed‐methods, 13 qualitative, and 4 are methodological (Table B3). The studies using mixed‐methods generally combine participatory workshops with sectoral or multi‐sectoral stakeholders or experts, and afterward integrate the results with modeling.^[^ ^100^, ^101^, ^102^, ^103^, ^104^, ^105^ ^]^ For instance, techniques such as foresight analysis including scenario planning. Some methodologies such as impact assessment and scenario planning emerged in this category, although with only few examples.^[^ ^106^, ^107^ ^]^ This is because the articles in this category generally use less frequently the concept of storyline, using instead the terms narratives or scenarios, as they could be considered to be at a later stage of the process.^[^ ^19^ ^]^) or landscape visualizations may be used during workshops or focus groups (see, e.g., Refs. [100, 108]). Several other studies used interviews and surveys in addition to workshops.^[^ ^79^, ^109^, ^110^, ^111^, ^112^, ^113^, ^114^, ^115^, ^116^, ^117^, ^118^ ^]^ Lippe and colleagues,^[^ ^110^ ^]^ for instance, used coupled models and participatory focus group discussions with participatory appraisal techniques to construct and validate storylines and the subsequent scenario storylines. Other interactive forms are games or agent‐based models.^[^ ^64^, ^119^ ^]^

Some of these studies involve stakeholders selectively at certain stages of the process. For instance, scholars may opt for involving stakeholders to estimate model parameter values^[^ ^109^ ^]^ or to evaluate outcomes by using surveys at the end of the process.^[^ ^108^ ^]^ Uniquely qualitative methods include Ahmed and colleagues^[^ ^120^ ^]^ who used questionnaires and interviews, designing four storylines/scenarios (the terms were used interchangeably, with only two mentions of the former). Participatory workshops without integrating modelling are found in several studies.^[^ ^115^, ^121^, ^122^, ^123^, ^124^, ^125^ ^]^ Mahlkov and colleagues^[^ ^126^ ^]^ used expert interviews and constellation analysis workshops. Finally, there are also methodological articles, such as Lempert,^[^ ^127^ ^]^ Maier et al.^[^ ^128^ ^]^ and Mallampalli et al.^[^ ^129^ ^]^ Mokrech et al.^[^ ^130^ ^]^ used multicriteria analysis and Groves et al.^[^ ^131^ ^]^ applied a robust decision‐making approach, which is based on scenario planning methodology (similarly as in^[^ ^106^, ^107^ ^]^).

#### 3.2.3 Summary of the Physical Climate Storylines

The PCS approach started around 2010. The approach is different from other scenario‐based concepts as it tries to represent uncertainty in the physical domain of climate change.^[^ ^18^ ^]^ Hazeleger et al.,^[^ ^132^ ^]^ for instance, introduced storylines to the physical aspects of climate change to study its drivers and their effects on future weather, calling the approach “tales of future weather”. It is a similar approach to mapping historical data onto the future climate while changing boundary conditions, considering compounding weather events and creating unprecedented events, such as the work seen in several studies.^[^ ^133^, ^134^, ^135^, ^136^ ^]^ These authors argue that storylines can investigate specific events more thoroughly, and their results can be used more easily compared to traditional climate model ensembles.^[^ ^132^ ^]^ At the same time, Trenberth et al.^[^ ^137^ ^]^ proposed a different framing to deal with dynamically driven extremes as opposed to conventional event attribution, focusing on what thermodynamic drivers impacted the specific event and how this would be altered due to climate change in the future.

In 2016, Shepherd unified these approaches and proposed the storyline approach, further refined in 2018 to “physically self‐consistent unfolding of past events, or of plausible future events or pathways”.^[^ ^18^ ^]^ PCS differs from the conventional frequentist approach that attaches probabilities to changes in extreme events when comparing a world without and with climate change, which is behind the concepts of counterfactual research and extreme event attribution. However, changing the framing of the extreme event attribution studies has caused a lot of debate in the physical science community.^[^ ^138^, ^139^ ^]^ Specifically, PCS focuses on understanding plausible futures that can be developed from investigating drivers of extreme events. The literature on PCS asserts that, depending on context, preferences and values, the conditionality of the storyline approach allows for the generation of multiple different storylines that could cover both type 1 (false discovery) and type 2 (missed warning) errors.^[^ ^140^ ^]^

Shepherd et al.^[^ ^18^ ^]^ showed four benefits of simulating the future in this fashion, which according to the author, stem from PCS being driven by a post‐normal science mindset, accepting that the future is unpredictable, evidence is contested, and a plurality of pathways are possible.^[^ ^141^ ^]^ Accordingly, the authors mention how storylines might improve risk awareness due to the focus on episodic memory (reliving experiences), using people's risk perception to simulate what might happen. Second, it might strengthen decision‐making by choosing plausible storylines over probable ones. The rationale was that PCS could enable the investigation of possible outcomes, which the authors believed fit the decision‐maker context better than traditional model ensemble averages. Third, instead of mixing the uncertainty of two main components of atmospheric response to its warming, thermodynamic and dynamic, they could be studied separately. Fourth, the boundaries of plausibility can be explored by improving the physical understanding of atmospheric processes and the drivers affecting them.

Several methodological directions can be taken to use the storyline approach. In general, large model ensembles with multiple members are used to explore climate simulations outside the historical data range and perturb drivers to create a plausible storyline.^[^ ^142^ ^]^ In addition, PCS can also be implemented through the use of causal networks,^[^ ^143^ ^]^ climate process chains,^[^ ^30^ ^]^ flow analogues,^[^ ^144^ ^]^ modeling chains,^[^ ^145^ ^]^ and multiple lines of evidence,^[^ ^45^ ^]^ depending on the spatial scale of interest. For example, local flood storylines are constructed differently than storylines of precipitation patterns in the Mediterranean basin. Due to the physical climate component, most articles focus on meteorological^[^ ^146^ ^]^ or hydrological^[^ ^147^ ^]^ impacts of climate change, while there has been some crossover into the agricultural sector.^[^ ^148^ ^]^ More details are provided in the subcategories below.

Since the introduction of the PCS approach, its application has expanded and adapted steadily. Its uptake by climate scientists was helped by the frontrunner studies with alternative approaches to climate prediction before the PCS storyline concept was introduced.^[^ ^149^, ^150^, ^151^ ^]^ From the original PCS, our review determined three subcategories based on the purpose and application of the storyline, its temporal and spatial scale, and specific methodologies. Of the original 57 PCS articles, we selected the articles with a fundamental background in the storyline approach (54), identifying the three main subcategories: Extreme event (41), Climatological trend (10) and Equilibrium climate sensitivity (3) storylines. Shepherd, 2016; Winsberg et al., 2020).

##### Extreme Events

**Figure 5:**
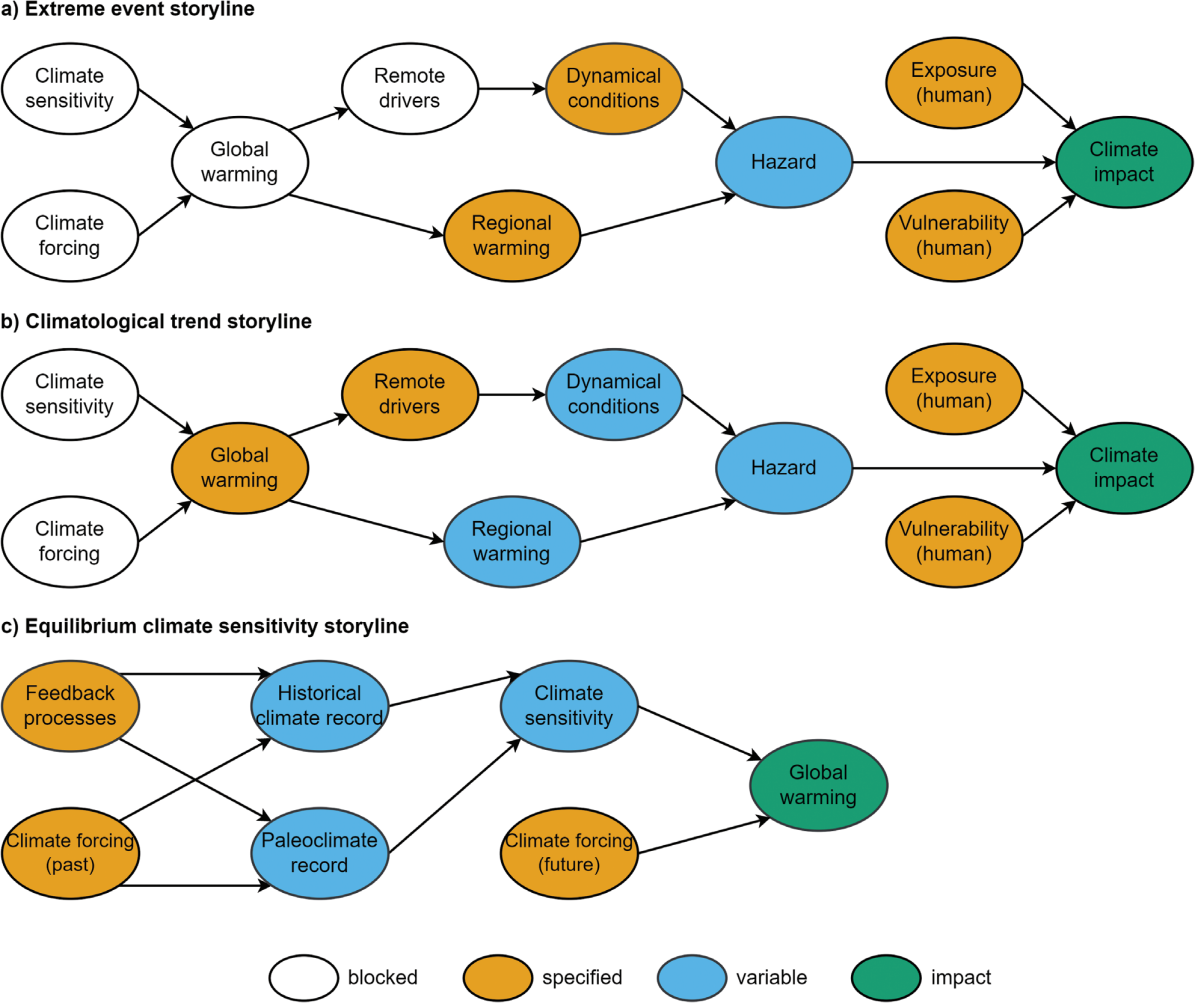
PCS subcategories are based on distinct choices in methodology, climate phenomena, and scale. Blocked (white) states are not taken into account, while the specified conditional states (orange) and the variables (blue) determine the impact (green) of the PCS storyline. a) Extreme event storylines focus on local/regional events and require dynamical conditions and regional warming to be specified to determine future hazards. b) Climatological trend storylines take on large‐scale processes where global warming and remote drivers are specified to investigate regional warming and dynamical conditions. c) Equilibrium climate sensitivity storylines focus on global warming effects and Earth's sensitivity to a doubling in CO_2_ and therefore specify climate forcings (past and future) and feedback processes. Original version from Shepherd,^[^ ^152^ ^]^ adapted with permission.^[^ ^41^, ^44^ ^]^ Copyright 2021, 2020, Cambridge University Press, John Wiley and Sons.

By far the most popular category in PCS, extreme event attribution and projection studies using the storyline approach category makes up for over 70% of total PCS publications. Unlike conventional probabilistic climate change methodology, PCS can focus on exploring the plausibility of a future extreme event and how it will be affected by climate change. As a complementary approach to risk‐based assessments, this could be useful for raising public awareness and studying climate impacts on smaller scales where dynamic effects (i.e., related to atmospheric and oceanic circulation, which are less robust in observations, theory, and models) are more important than thermodynamics (i.e., including continental or basin‐scale averages such as sea level, surface air temperature or sea ice extent, for instance, which are robust in observations, theory, and models).^[^ ^152^ ^]^ However, conditioning the physical aspects of climate change to local or regional contexts is crucial to make extreme weather impacts more applicable for decision‐makers and modelers (**Figure** **5**). Shepherd^[^ ^152^ ^]^ also argues it can help to question the effects of interventions across plausible futures while providing a benchmark based on historical events.

Extreme event attribution and projection studies deal with a wide range of extreme future events that hypothetically could occur, so‐called counterfactuals.^[^ ^153^ ^]^ The climate phenomena studied include extreme precipitation events, floods and hurricanes, heatwaves, droughts, and compound events. Model ensembles are frequently used to investigate these extremes,^[^ ^154^, ^155^, ^156^ ^]^ and the analogs created through simulations can be modified to include the effects of climate change.^[^ ^144^, ^148^, ^157^, ^158^ ^]^ Other studies in this subcategory explore climate impacts with pseudoglobal warming^[^ ^159^, ^160^, ^161^ ^]^ and with the adjustment of dynamic model variables.^[^ ^162^, ^163^, ^164^ ^]^ Other studies convert the climate model to an impact model using a modeling chain, fitting the specific policy context.^[^ ^145^, ^147^, ^165^ ^]^

In this subcategory, there is the causal networks extension. In it, storylines represent different pathways in a causal network that try to link climate drivers to impacts.^[^ ^143^ ^]^ Although such networks simplify the climate system, counterfactual outcomes are considered through soft‐bayesian approaches to combine the plausible data with probabilistic values to adhere to policy norms.^[^ ^152^ ^]^ Based on the research goal, the network's context can be shifted to target specific causal elements and benefit from the transparency of conditional hypotheses.^[^ ^10^ ^]^ This extension doubles down on the forensic investigation aspect of storylines which treats every extreme event as unique and follows a “case study” methodology.^[^ ^43^, ^140^ ^]^

##### Climatological Trends

This subcategory contains 15% of the total PCS corpus. Similar to extreme event frequencies, magnitudes, and impacts, future trends in mean precipitation and temperature can be impacted by climate change. The primary purpose of these studies is to investigate the remote drivers contributing to changes in atmospheric circulation. In particular, plausible responses of remote drivers of seasonal precipitation patterns are conditioned to estimate the drying and wetting patterns in a future climate associated with a particular atmospheric circulation change. This focus is motivated by the predominant role of atmospheric circulation causing uncertainties in the changes of precipitation.^[^ ^166^, ^167^ ^]^ The scale of these adjustments increases compared to extreme event studies, as the remote drivers cover regional^[^ ^168^ ^]^ to hemispheric^[^ ^169^ ^]^ ranges (Figure 5).

Phenomena treated in climatological trend attribution and projection range from seasonal drying and wetting patterns to tropospheric circulation modes and stratospheric polar vortexes. They can include windiness, air stagnation, storm tracks, and moisture balances. Following a mathematical approach, atmospheric variables from model ensembles are often perturbed to consider the atmospheric processes with and without the effects of climate change. A physical approach involves linear regression combined with a process‐based understanding of the remote drivers and possible stratosphere‐troposphere coupling. Among the considered remote drivers, the analyzed papers used tropical amplification and stratospheric vortex,^[^ ^146^, ^169^, ^170^ ^]^ atmospheric moisture,^[^ ^156^ ^]^ atmospheric circulation modes and sea surface temperatures,^[^ ^168^ ^]^ and tropical versus arctic warming.^[^ ^171^ ^]^ To extend the analysis, Mindlin et al.^[^ ^172^ ^]^ studied the time‐evolving remote driver response to greenhouse gases and ozone changes.

##### Equilibrium Climate Sensitivity

Compared to the other two subcategories, the number of articles that use the PCS approach to investigate global climate sensitivity is very limited. Of the 57 PCS articles, only three articles investigated the climate sensitivity to a doubling of carbon dioxide. In the context of these sensitivities to global warming, storylines were constructed and refuted to reduce the plausible bounds of the earth's temperature response.^[^ ^45^, ^46^, ^173^ ^]^ Reasoning by refutation allows to omit implausible storyline whenever the evidence disagrees, consequently building greater confidence in the temperature range increase attributed to climate change.^[^ ^45^ ^]^

The methodology followed by two articles is called the multiple lines of evidence approach. Multiple sources of scientific evidence are investigated, and their changes have to be independent and in agreement to constrain the bounds of climate sensitivity.^[^ ^46^ ^]^ Model feedback processes, the historical climate records, and the paleoclimate are recognized as possible evidence of the earth's temperature response^[^ ^45^ ^]^ (Figure 5). The bounds are constrained when the conditions for a particular sensitivity response are met by independent evidence. However, the original bounds are maintained when one or more conditions cannot be met. Even though this subcategory is dedicated to the impacts on global climate, building conditional hypotheses to dissect past climate and construct the future has many overlaps with the other PCS subcategories. Similar to the causal networks mentioned earlier, the lines of evidence can be combined in a Bayesian network to quantify the confidence range and attach probabilities.

### 3.3 Critiques

In the course of the review, we also looked at the critiques received by the different approaches that use storylines in climate‐related science, which we summarize next.

The weakness of discourse‐analytical approaches, as seen by some authors, comes from the fact that discourse analysis is context‐bounded, often hampering the scalability of findings as observed in the fields of climate change adaptation policy and vulnerability studies.^[^ ^38^, ^103^, ^174^ ^]^ However, these approaches are more attuned to different forms of expertise and their importance, and how agents' perceptions, interests, and reactions may influence efforts in climate policy.^[^ ^51^ ^]^

Concerns regarding scenario methodologies include their lack of touch with reality (see, e.g., Refs. [88, 175]), and the fact that they are not sufficiently quantitative,^[^ ^176^ ^]^ or they lack ways to prove their consistency.^[^ ^96^, ^97^ ^]^ Another critique is related to transparency in the way this approach assembles the storylines.^[^ ^177^ ^]^ However, transparency is crucial when deciding the type and number of storylines, which is not a value‐free choice, as interests can play a big role in what we envisage as a possible future.^[^ ^178^, ^179^ ^]^ Moreover, whilst participatory approaches have been identified as a criterion to assess the quality of storylines,^[^ ^19^ ^]^ relative few studies use such methodologies as many studies depart from the SSPs, which were initially criticized for not consulting a broader academic community and all relevant actors, including an uneven representation of the Global South.^[^ ^73^, ^91^, ^180^ ^]^ We can expect that the physical climate storylines literature might face this same critique in the future, as PCS rarely incorporate other disciplines or stakeholders in the decisions about the components used to assemble storylines, as revealed by our review.

Physical climate storyline scholars add to these critiques by emphasizing that traditional (RCP)‐SSP scenarios frame the future climate as “singular, definitive”, acknowledging uncertainty but not exploring it fully. On the contrary, PCS scholars argue that their methodology allows for a “plural, conditional” framing of uncertainty.^[^ ^10^ ^]^ The idea of conditional analysis is useful because of their applicability to adaptation, also because it enables treating three sources of uncertainty that affect regional climate change projections which are of different nature and were mistreated in mainstream approaches.^[^ ^44^ ^]^ However, PCS were in turn regarded with skepticism from the traditional event attribution community (see Ref. [143]). Introducing the storyline approach to the detection and attribution branch of extremes was perceived as rendering former risk‐based approaches unscientific and incorrect. However, Lloyd and Oreskes^[^ ^143^ ^]^ argue that there is no right or wrong approach and depending on the context of relative risk, both approaches can be complementary. Finally, and as commented previously, despite the claims to be a useful tool for decision‐making, our results show that PCS remain in the physical climate science domain, allowing little interaction with stakeholders or integration with socio‐economic components, with a few exceptions discussed in the Discussion section.

## 4 Discussion

Storyline‐reliant approaches can be valuable tools for supporting decision‐making processes and making climate evolution more tangible to societal agents.^[^ ^19^, ^127^, ^181^ ^]^ Due to their flexibility, they allow for methodological innovations encouraging creativity in the scientific undertaking. However, the body of literature on storylines is vast, and navigating it has become more challenging due to the plurality of uses. In this article, we explored how the concept of storylines has been treated in the three identified approaches which are being used by often disconnected scholarly communities. Despite departing from a similar willingness to understand and bring climate science and policy closer, “storylines” in each approach consist of different objects and are studied with a different set of methods. In scenario studies, storylines are the qualitative elements from which socio‐economic pathways may depart and be linked with physical climate science through modeling. On the contrary, storylines in PCS rather characterize the whole conditional process chain of physical climate variables evolution but tend to disregard socioeconomic factors. Then again, discourse‐analytical approaches focus on identifying the storylines that form the discourses that are prevalent in climate‐related policy‐making. Methodologically, it is illustrative that the approaches show clustering tendencies in the use of methods, with over 50% of scenario‐based approaches using solely quantitative modeling techniques, most discourse‐analytical approaches using qualitative methods, and 75% of PCS using event attribution and climate projections.

In this section we depart from the methodologies we have observed in each approach, as well as the strengths and critiques identified in the literature, to answer the second research question: “*how can cross‐pollination among these communities enable the assembly of more useful and usable climate‐related storylines?*”.

Starting with scenario‐based approaches, there are certain good practices that could address the critiques of lacking either touch with reality or transparency, not being sufficiently quantitative or qualitative, proving consistency or missing participatory approaches. For instance, Mitter and colleagues^[^ ^19^ ^]^ present a 9‐stage protocol that aims to meet quality criteria during the process of producing storylines. Following the staged protocol could already address issues of transparency. Equally, depending on the team and stakeholders involved, bringing the “storylines” closer to policy‐making would become easier. However, choosing a team of scientists as well as the type of stakeholders involved is one of the aspects that could gain from cross‐pollination with other practices.

Scientists from different disciplines working together in scenario methodologies is already common practice. However, these practices do not always reach the full scope of working equally together that transdisciplinarity implies or might leave out *certain* disciplines or expertise. In this sense, DAA shows that social sciences and humanities are not a homogeneous expertise. A range of methods and perspectives put forward by a trained economist would diverge greatly from those proposed by an anthropologist.^[^ ^4^ ^]^ Moreover, these experts can diverge in ontological and epistemic positions, which could also be relevant to acknowledge.^[^ ^22^ ^]^ Hence, a comprehensive engagement of scientists with different expertise acquainted with discourse‐analytical approaches could make transdisciplinarity more approachable.

Besides, a discourse analyst could identify if the considered range of storylines is pluralist, which depends on the beliefs and visions of the scientists involved, and on the range of stakeholders that participate.^[^ ^71^ ^]^ For instance, Drunen et al.^[^ ^182^ ^]^ described the characteristics of the main IPCC‐related storyline archetypes as: economic optimism; reformed markets; global sustainable development; regional sustainability; regional competition; business‐as‐usual. A discourse analyst could argue that the majority of these storylines depart from an economic growth perspective (ranging from slow to rapid, and more to less green), thereby ignoring alternative societal pathways^[^ ^183^, ^184^, ^185^ ^]^ for more on these debates see.^[^ ^186^ ^]^

Opting for participatory processes, i.e., including stakeholders, in the design of storylines can similarly address some of the identified critiques. But similarly, participatory scenarios can lack credibility depending on the type of mental models represented by the stakeholders. Thus, scientists should be aware that choosing stakeholders with whom to engage in the process is not a value‐free choice and should be approached carefully and systematically [on stakeholder selection guidance see Refs. [187, 188]. Involving a wide range of stakeholders can also help deal with the challenge such as storyline convergence – when the produced storylines are too similar^[^ ^106^ ^]^—which might occur when there is homogeneity of actors involved.

"Physical climate storylines" could benefit from stakeholder engagement and involvement of interdisciplinary teams. The PCS literature aims to produce storylines that are societally relevant because climate risks are reframed and conditioned to fit the decision‐making context.^[^ ^152^ ^]^ However, PCS are the group of studies that least aligns with transdisciplinary requirements or participatory process designs, which can distance them from decision‐making contexts. This is not surprising given that many of the studies are very technical, requiring expertise in climate science and processes, while most articles only provide hazard‐related variables (e.g., temperature and precipitation extremes). Among the exceptions are articles where the modeling chain of the storyline reaches the decision‐making context^[^ ^147^, ^189^ ^]^ or where an impact metric of an extreme event is calculated.^[^ ^148^ ^]^ Authors that opt for PCS do not need to involve stakeholders during all the process, but equally as observed in some of the scenario literature, stakeholders could be engaged in selected stages of assembling the storyline, such as for instance in the choice of the relevant extreme events or at the final stage to discuss the results.

Some of the most recent studies in the PCS literature already introduced such participatory processes. For instance, Jack and colleagues^[^ ^31^ ^]^ conducted workshops with researchers from different disciplines (expert judgment) and with stakeholders. Participatory methodology in these PCS examples highlights the importance of personal experience for building storylines and the consideration of multiple plausible futures. However, instead of solely investigating physical science, attention may be directed to transdisciplinary work, coproducing storylines that convey climate risks to local communities and decision‐makers. Similarly, the serious games approach by Penney et al.^[^ ^190^ ^]^ used the extreme event attribution to create multiple counterfactual future worlds with interdisciplinary groups of disaster researchers. Yet another example of an alternative approach is expert judgment, as used by Dessai et al.^[^ ^191^ ^]^ for constructing six climate storylines based on two drivers of regional precipitation changes for a South Indian watershed. The authors captured the plausible range of future precipitation using a combination of local and foreign climate scientists’ expertise. Although far from mainstream, these recent remakes of PCS illustrate a pathway that this approach could be taking in the future.

In short, both SB and PCS could benefit from discourse‐analytical approaches’ practice in organizing inclusive, participatory workshops, adapting the discussions, and identifying a broad range of stakeholders with different knowledge expertise and backgrounds. This would address legitimacy concerns. Enlarging also the range of quality criteria to assess storylines considered could in fact appease some of the critiques in PCS.^[^ ^139^ ^]^ In this sense, storyline‐relevant quality criteria may well include considerations regarding vertical and horizontal consistency, salience, legitimacy, or richness and creativity and not only “plausibility” as it is usually the case in PCS.^[^ ^19^, ^71^ ^]^

Finally, discourse‐analytical approaches may lack scalability of findings, which, alternatively, the scenario‐based literature shows as one of its strengths. DAA are context‐bounded and, for this reason, feasibility of scalability or transferability is on case‐by‐case basis, also depending on the extent of the policy process the storyline encompasses. An increasing presence of mixed‐methods among such studies could contribute to this endeavor. Especially, DAA could gain from the use of simulations to understand the future perspectives of prevalent discourses. For this, increasing the collaboration with simulation‐prone disciplines from both social and physical sciences could enrich some of the DAA projects.

Overall, each of the identified storyline‐reliant approaches utilizing storylines can greatly gain from the strengths that the alternative approaches provide.

## 5 Conclusions

Storylines are a useful tool to inform and support climate change adaptation, mitigation, and risk assessment processes. This paper provides an overview of the state‐of‐the‐art of climate‐related storylines through a semi‐systematic literature review. This has resulted in a classification of the vast and heterogenous literature into three categories, giving a good starting point for those interested in delving into the different storyline approaches and methodologies. Additionally, we identify valuable elements from each approach that could be incorporated into the others, i.e., a cross‐pollination of good practices from the three approaches which could lead to more useful storylines for decision‐making. Good practices for the development of storylines may favor the involvement of different disciplines and non‐academic stakeholders in key stages of the process, as well as have a preference for mixed‐methods. Thus, not only would modelers be involved in the process of constructing storylines, as it is common practice now (e.g., physical climate scientists and economists), but also other experts and disciplines that opt for different methods and depart from other theoretical or meta‐theoretical frameworks. Likewise, good practices should also welcome stakeholders representing a variety of perspectives and interests. Storylines can this way become more realistic, actionable, and usable to start reducing the climate action gap.

To conclude, we would like to encourage scholars interested in the construction of storylines to be open to methodologies applied by other schools of thought. We believe that, by pointing at the strengths of each of the current three main approaches, this article paved the way for improving and enriching storyline construction practices, as well as for scaling up their impact. Such cross‐pollination of good practices can best ensure that storylines meet the quality standards acknowledged by the scholarly community working in climate‐related science.

## Conflict of Interest

The authors declare no conflict of interest.

## Supporting information

Supporting Information
