# Supplementary material for: Assembling the climate story: use of storyline approaches in climate‐related science
Source: Glob Chall. 2023 May 23;7(7):2200183. doi: 10.1002/gch2.202200183 (PMC10362102; doi:10.1002/gch2.202200183)
Supplement: Supplementary file 1 — Supporting Information [file GCH2-7-2200183-s001.pdf]

Global
Challenges
Open Access

Supporting Information

for *Global Challenges.*, DOI 10.1002/gch2.202200183

Assembling the climate story: use of storyline approaches in climate-related science

Eulàlia Baulenas, Gerrit Versteeg, Marta Terrado, Julia Mindlin and Dragana Bojovic*

Supporting Information Section 1: BACKWARD CITATIONS

To conduct backward citation, the final selection is trimmed to 265 articles with a valid DOI. Then, the articles were imported into https://www.lens.org/ to identify all the Lens IDs and acquire the references of each article. The data was exported in CSV format and uploaded to the VOSviewer, a bibliographical mapping tool. Here, we create a map based on bibliographical data, select the bibliographic coupling of documents option and choose the full counting method. Before plotting the map, we only displayed connected articles and discard unconnected ones. Therefore, only 248 of the original 270 articles were displayed, partly explained by the articles needing DOIs and references.

Table A1: Backwards citation results, giving the top 5 articles of each main category with the most frequently shared references

#	Authors	Title	Year
Scenario-based approaches			
25	O'Neill et al.	A new **scenario** framework for climate change research: the concept of shared socioeconomic pathways	2013
24	Nakicenovic et al.	Special report on emissions **scenarios**	2000
23	Van Vuuren et al.	The representative concentration **pathways**: an overview	2011
21	O'Neill et al.	The roads ahead: **Narratives** for shared socioeconomic **pathways** describing world futures in the 21st century	2017
20	Moss et al.	The next generation of **scenarios** for climate change research and assessment	2010
Physical climate storylines			
32	Shepherd et al.	**Storylines**: an alternative approach to representing uncertainty in physical aspects of climate change	2018
18	Hazeleger et al.	**Tales** of future weather	2015
18	Shepherd	Atmospheric circulation as a source of uncertainty in climate change projections	2014
16	Shepherd	**Storyline** approach to the construction of regional climate change information	2019
15	Zappa & Shepherd	**Storylines** of Atmospheric Circulation Change for European Regional Climate Impact Assessment	2017
Discourse-analytical approaches			
30	Hajer	The politics of environmental **discourse**	1995
14	Dryzek	The Politics of the Earth: Environmental **Discourses**	1997
13	Hajer & Versteeg	A decade of **discourse** analysis of environmental politics: Achievements, challenges, perspectives	2005
8	Bulkeley	**Discourse** Coalitions and the Australian Climate Change Policy Network	2000
7	Hajer	Doing **Discourse** Analysis: Coalitions, Practices, Meaning	2006

Supporting Information Section 2: LIST OF REFERENCES.

A. Constructivist approaches

**Table A1:** List of studies using ADA
(Aldunce et al., 2014, 2015; Anshelm & Hansson, 2014; Asayama & Ishii, 2017; Ayeb-Karlsson, 2020a, 2020b personal in-depth life histories / storytelling methodology; Bastakoti & Davidsen, 2017; Blum & Lövbrand, 2019; Boas & Rothe, 2016; Cherry et al., 2015; Cotton et al., 2014; Curran, 2012; Holmgren et al., 2019; Hurri, 2020; Kangas, 2019; Karhunmaa, 2016; Leung et al., 2018; Lovell et al., 2009; Lucas & Warman, 2018; Markard et al., 2021 with discourse network analysis, analysing the evolution of discourse coalitions; Mather-Gratton et al., 2021; Matthews, 2015; Matthews & Marston, 2019; Mayrhofer & Gupta, 2016 also merging with discourse institutionalism, but using the definition of Hajer of storylines; Melo et al., 2014; Morton, 2021; Neubauer & Gunster, 2019; Newing, 2009; Nielsen, 2016; Schenkel, 2000; Taylor et al., 2014; Teräväinen, 2010; Toke, 2013; van Eck & Feindt, 2021; Vogel et al., 2021; Zelli et al., 2019)

B. Hybrid scenarios dataset

Table B1. Literature from the set of studies departing from IPCC-based scenarios

Sectoral applications

**Sector**	**Studies**
**Agriculture**	(Avnery et al., 2011; Bocchiola et al., 2013; Engström et al., 2016; Graux et al., 2012 on lifestock; Kros et al., 2015; Nechifor & Winning, 2019; Nicholls, 2004; Nunes et al., 2017; Rankinen et al., 2013; Tripathy et al., 2012)
**Aviation**	(Gudmundsson & Anger, 2012)
**Biodiversity**	(Fronzek et al., 2012; Merino et al., 2010; Politti et al., 2014; Thuiller et al., 2005, 2006)
**Carbon and other GHG**	(Fenhann, 2000; Müller et al., 2007; Riahi & Roehrl, 2000; Sankovski et al., 2000; Shindell et al., 2008; Sjøeng et al., 2009; Wheeler et al., 2013; Zaehle et al., 2007)
**Economy**	(Dellink et al., 2017; Delzeit et al., 2021; Goklany, 2005)
**Energy**	(Creutzig et al., 2012; Dias et al., 2016; Hainsch et al., 2022; Jalilzadehazhari et al., 2021; Mirasgedis et al., 2007; Rogner et al., 2008; Samsuri et al., 2018)
**Food**	(Alessandrini & Bodirsky, 2020 on food/dietary "qualitative storyline" megatrends developed along the SSPs)
**Forest**	(Daigneault et al., 2019; Dale et al., 2010; Ding et al., 2012; Eggers et al., 2008; Garzón et al., 2008; Morin et al., 2008; Morin & Chuine, 2014; Obersteiner et al., 2006; Poulter, Aragão, et al., 2010; Poulter, Hattermann, et al., 2010)

**Health**	(Sellers & Ebi, 2018)
**Land use**	(Mancosu et al., 2015; Popp et al., 2017; Rounsevell et al., 2005; Rounsevell & Metzger, 2010; Samson et al., 2018; van Vuuren et al., 2017)
**Production**	(Pauliuk et al., 2021)
**Sanitation**	(Hofstra & Vermeulen, 2016)
**Sea level**	(Lin et al., 2020; Nauels et al., 2017 linking sea level rise with socioeconomic indicators from the SSP)
**Tourism**	(Becken et al., 2015 using interviews to fit in the data for the useful climate variables to use. Departing from A2 emission scenarios with coproduction.)
**Water**	(Arnell, 2004; Booth et al., 2016 with coproduction to design the "scenario storylines" which sometimes it is mixed with the word "narrative"; Confortola et al., 2013; Couture et al., 2018; Freund et al., 2017; Hutchins et al., 2018; Jorda-Capdevila et al., 2019; Kim et al., 2013; Mack et al., 2019; Molina-Navarro et al., 2018; Parish et al., 2012; Semadeni-Davies et al., 2008 with coproduction, but without explaining the process; Sheshukov et al., 2011; Strasser et al., 2019; Yuan et al., 2005).
**Several**	(Arnell et al., 2004; Serpa et al., 2015, 2017)

Table B2. Literature from the set of studies not departing from IPCC-based scenarios

Modelling and simulation literature

**List of studies using modelling or simulation techniques**
(One exception to the use of modelling is found in Alisjahbana & Busch, 2017 who use descriptive statistics for trends in Indonesia assessing "storylines" in terms of commitments for achieving the carbon targets; Almeida et al., 2018; Bauer et al., 2019, "the scenarios are built on consistent storylines that describe plausible developments of climatic and socioeconomic factors"; Bayer et al., 2021 on model evaluation; Berckmans et al., 2019; Bierwagen et al., 2010; Bonfante et al., 2015; Château, 2014 on EU transitions to low carbon; Ding et al., 2016 with economic and physical climate variables, on EU forestry; Drake et al., 2021 on GHG; Gerst et al., 2013 on scenario discovery (for policy outcomes) using agent-based modelling; Henriques, 2007 on water resource management; Honjo et al., 2021; Immerzeel, 2008; Jiang, Masui, et al., 2000; Jiang, Morita, et al., 2000; Knevels et al., 2020; Leimbach et al., 2016; Melsen et al., 2018; Mori, 2000a, 2000b; Mullens & McPherson, 2019; Nussbaumer et al., 2014; Pais et al., 2020; Poulter, Aragão, et al., 2010; Poulter, Hattermann, et al., 2010; Rankinen et al., 2019; Rogge et al., 2020; Schaldach et al., 2017; Spencer & Dubash, 2022; Wang et al., 2013).

Supporting Information Section 3: LIST OF KEYWORDS AND DEFINITIONS

Discourse-analytical approaches

Storyline	"a condensed form of narrative in which metaphors are used" (Hajer, 2006, p. 69)
Narratives	"a story ascribing meaning to social or physical phenomena by connecting a sequence of events and actions in a plot, including, excluding, and emphasizing problems, actors, and events and, thus providing an interpretation of who or what is significant (c.f. Hardy et al. 1968, Hajer 1995, Feldman 2004 Leipold, 2021, p. 1047).
Discourse	"Ensemble of ideas, concepts, and categories through which meaning is given to phenomena. Discourses frame certain problems; that is to say, they distinguish some aspects of a situation rather than others." (Hajer, 2006, p. 45)
Discourse coalition	"Group of actors who share a social construct" (Hajer, 2006, p. 45)
Discourse institutionalization	"If a discourse is successful -that is to say, if many people use it to conceptualize the world- it will solidify into an institution, sometimes as organizational practices, sometimes as traditional ways of reasoning." (Hajer, 2006, p. 46)
Frame	"a central organising idea or *story line* that provides meaning to an unfolding strip of events, weaving a connection among them. The frame suggests what the controversy is about, the essence of the issue" (c.f. Gamson and Modigliani 1987:143, in Asplund et al., 2013, italics added).

Scenario-based approaches definitions according to the IPCC glossary

Storyline	A narrative description of a scenario (or family of scenarios), highlighting the main scenario characteristics, relationships between key driving forces and the dynamics of their evolution (IPCC, 2014).
Narratives	Qualitative descriptions of plausible future world evolutions, describing the characteristics, general logic and developments underlying a particular quantitative set of scenarios. Narratives are also referred to in the literature as 'storylines' (IPCC, 2018).
Scenario	A plausible description of how the future may develop based on a coherent and internally consistent set of assumptions about key driving forces (e.g., rate of technological change (TC), prices) and relationships. Note that scenarios are neither predictions nor forecasts, but are used to provide a view of the implications of developments and actions (IPCC, 2019).
Scenario storyline	A narrative description of a scenario (or family of scenarios), highlighting the main scenario characteristics, relationships between key driving forces and the dynamics of their evolution. Also referred to as 'narratives' in the scenario literature (IPCC, 2019).
Pathways	The temporal evolution of natural and/or human systems towards a future state. Pathway concepts range from sets of quantitative and qualitative scenarios or narratives of potential futures to solution-oriented decision-making processes to achieve desirable societal goals. Pathway approaches typically focus on biophysical, techno-economic, and/or socio-behavioural trajectories and involve various dynamics, goals, and actors across different scales. Examples include representative concentration pathways and shared-socioeconomic pathways (IPCC, 2019).

PCS definitions (*interpretated in text)

Two critical differences between PCS and scenario-based approaches are mentioned in Shepherd et al. 2018. The authors state that the constructed storylines differ from DAA because the focus is on the representation of uncertainty instead of the framing of discourse. And, because PCS aims to represent uncertainty in the physical aspect of climate change instead of the anthropologic domain, it does not share a similar framework to SB. Even though the terminology might be similar between the categories, each approach consists of a methodological framework that fits a specific purpose.

Storyline	A physically self-consistent unfolding of past events, or of plausible future events or pathways (Shepherd et al., 2018).
Narratives*	Causal network that anchors the storyline and represent uncertainty (Shepherd, 2016; Shepherd et al., 2018)
Scenario*	Can be regarded as storylines in which information on both socio-economic developments and climate change are combined in one narrative (2015).
Pathways*	Trends of future events, which could be long-lasting (Shepherd et al., 2018).
Tales*	See narratives

References

Aldunce, P., Beilin, R., Handmer, J., & Howden, M. (2014). Framing disaster resilience: The implications of the diverse conceptualisations of “bouncing back.” *Disaster Prevention and Management: An International Journal*, *23*(3). https://doi.org/10.1108/DPM-07-2013-0130

Aldunce, P., Beilin, R., Howden, M., & Handmer, J. (2015). Resilience for disaster risk management in a changing climate: Practitioners’ frames and practices. *Global Environmental Change*, *30*. https://doi.org/10.1016/j.gloenvcha.2014.10.010

Alessandrini, R., & Bodirsky, B. L. (2020). Food futures: Storylines of dietary megatrends along the Shared Socioeconomic Pathways (SSPs). *Proceedings of the Nutrition Society*, *79*(OCE2). https://doi.org/10.1017/s002966512000275x

Alisjahbana, A. S., & Busch, J. M. (2017). Forestry, Forest Fires, and Climate Change in Indonesia. *Bulletin of Indonesian Economic Studies*, *53*(2). https://doi.org/10.1080/00074918.2017.1365404

Almeida, C., Ramos, T. B., Segurado, P., Branco, P., Neves, R., & de Oliveira, R. P. (2018). Water quantity and quality under future climate and societal scenarios: A basin-wide approach applied to the Sorraia River, Portugal. *Water (Switzerland)*, *10*(9). https://doi.org/10.3390/w10091186

Anshelm, J., & Hansson, A. (2014). The Last Chance to Save the Planet? An Analysis of the Geoengineering Advocacy Discourse in the Public Debate. *Environmental Humanities*, *5*(1). https://doi.org/10.1215/22011919-3615433

Arnell, N. W. (2004). Climate change and global water resources: SRES emissions and socio-economic scenarios. *Global Environmental Change*, *14*(1). https://doi.org/10.1016/j.gloenvcha.2003.10.006

Arnell, N. W., Livermore, M. J. L., Kovats, S., Levy, P. E., Nicholls, R., Parry, M. L., & Gaffin, S. R. (2004). Climate and socio-economic scenarios for global-scale climate change impacts assessments: Characterising the SRES storylines. *Global Environmental Change*, *14*(1). https://doi.org/10.1016/j.gloenvcha.2003.10.004

Asayama, S., & Ishii, A. (2017). Selling stories of techno-optimism? The role of narratives on discursive construction of carbon capture and storage in the Japanese media. *Energy Research and Social Science*, *31*. https://doi.org/10.1016/j.erss.2017.06.010

Avnery, S., Mauzerall, D. L., Liu, J., & Horowitz, L. W. (2011). Global crop yield reductions due to surface ozone exposure: 2. Year 2030 potential crop production losses and economic damage under two scenarios of O3 pollution. *Atmospheric Environment*, *45*(13). https://doi.org/10.1016/j.atmosenv.2011.01.002

Ayeb-Karlsson, S. (2020a). ‘I do not like her going to the shelter’: Stories on gendered disaster (im)mobility and wellbeing loss in coastal Bangladesh. *International Journal of Disaster Risk Reduction*, *50*. https://doi.org/10.1016/j.ijdrr.2020.101904

Ayeb-Karlsson, S. (2020b). ‘When we were children we had dreams, then we came to Dhaka to survive’: urban stories connecting loss of wellbeing, displacement and (im)mobility. *Climate and Development*. https://doi.org/10.1080/17565529.2020.1777078

Bastakoti, R. R., & Davidsen, C. (2017). Framing REDD+ at national level: Actors and discourse around Nepal’s policy debate. *Forests*, *8*(3). https://doi.org/10.3390/f8030057

Bauer, B., Gustafsson, B. G., Hyytiäinen, K., Meier, H. E. M., Müller-Karulis, B., Saraiva, S., & Tomczak, M. T. (2019). Food web and fisheries in the future Baltic Sea. *Ambio*, *48*(11). https://doi.org/10.1007/s13280-019-01229-3

Bayer, A. D., Fuchs, R., Mey, R., Krause, A., Verburg, P. H., Anthoni, P., & Arneth, A. (2021). Diverging land-use projections cause large variability in their impacts on ecosystems and related indicators for ecosystem services. *Earth System Dynamics*, *12*(1). https://doi.org/10.5194/esd-12-327-2021

Becken, S., Zammit, C., & Hendrikx, J. (2015). Developing Climate Change Maps for Tourism: Essential Information or Awareness Raising? *Journal of Travel Research*, *54*(4). https://doi.org/10.1177/0047287514528286

Berckmans, J., Hamdi, R., & Dendoncker, N. (2019). Bridging the Gap Between Policy-Driven Land Use Changes and Regional Climate Projections. *Journal of Geophysical Research: Atmospheres*, *124*(12). https://doi.org/10.1029/2018JD029207

Bierwagen, B. G., Theobald, D. M., Pyke, C. R., Choate, A., Groth, P., Thomas, J. v., & Morefield, P. (2010). National housing and impervious surface scenarios for integrated climate impact assessments. *Proceedings of the National Academy of Sciences of the United States of America*, *107*(49). https://doi.org/10.1073/pnas.1002096107

Blum, M., & Lövbrand, E. (2019). The return of carbon offsetting? The discursive legitimation of new market arrangements in the Paris climate regime. *Earth System Governance*, *2*. https://doi.org/10.1016/j.esg.2019.100028

Boas, I., & Rothe, D. (2016). From conflict to resilience? Explaining recent changes in climate security discourse and practice. *Environmental Politics*, *25*(4). https://doi.org/10.1080/09644016.2016.1160479

Bocchiola, D., Nana, E., & Soncini, A. (2013). Impact of climate change scenarios on crop yield and water footprint of maize in the Po valley of Italy. *Agricultural Water Management*, *116*. https://doi.org/10.1016/j.agwat.2012.10.009

Bonfante, A., Monaco, E., Alfieri, S. M., de Lorenzi, F., Manna, P., Basile, A., & Bouma, J. (2015). Climate change effects on the suitability of an agricultural area to maize cultivation: Application of a new hybrid land evaluation system. *Advances in Agronomy*, *133*. https://doi.org/10.1016/bs.agron.2015.05.001

Booth, E. G., Qiu, J., Carpenter, S. R., Schatz, J., Chen, X., Kucharik, C. J., Loheide, S. P., Motew, M. M., Seifert, J. M., & Turner, M. G. (2016). From qualitative to quantitative environmental scenarios: Translating storylines into biophysical modeling inputs at the watershed scale. *Environmental Modelling and Software*, *85*, 80–97. https://doi.org/10.1016/j.envsoft.2016.08.008

Château, B. (2014). Spacecraft: A steady progress scenario for the EU. *Futures*, *58*. https://doi.org/10.1016/j.futures.2014.01.002

Cherry, C., Hopfe, C., MacGillivray, B., & Pidgeon, N. (2015). Media discourses of low carbon housing: The marginalisation of social and behavioural dimensions within the British broadsheet press. *Public Understanding of Science*, *24*(3). https://doi.org/10.1177/0963662513512442

Confortola, G., Soncini, A., & Bocchiola, D. (2013). Climate change will affect hydrological regimes in the Alps. *Revue de Géographie Alpine*, *101–3*. https://doi.org/10.4000/rga.2176

Cotton, M., Rattle, I., & Alstine, J. van. (2014). Shale gas policy in the United Kingdom: An argumentative discourse analysis Updated version forthcoming in Energy Policy. *Energy Policy*, *73*.

Couture, R. M., Moe, S. J., Lin, Y., Kaste, Ø., Haande, S., & Lyche Solheim, A. (2018). Simulating water quality and ecological status of Lake Vansjø, Norway, under land-use and climate change by linking process-oriented models with a Bayesian network. *Science of the Total Environment*, *621*. https://doi.org/10.1016/j.scitotenv.2017.11.303

Creutzig, F., von Stechow, C., Klein, D., Hunsberger, C., Bauer, N., Popp, A., & Edenhofer, O. (2012). Can bioenergy assessments deliver? *Economics of Energy and Environmental Policy*, *1*(2). https://doi.org/10.5547/2160-5890.1.2.5

Curran, G. (2012). Contested energy futures: Shaping renewable energy narratives in Australia. *Global Environmental Change*, *22*(1). https://doi.org/10.1016/j.gloenvcha.2011.11.009

Daigneault, A., Johnston, C., Korosuo, A., Baker, J. S., Forsell, N., Prestemon, J. P., & Abt, R. C. (2019). Developing Detailed Shared Socioeconomic Pathway (SSP) Narratives for the Global Forest Sector. *Journal of Forest Economics*, *34*(1–2). https://doi.org/10.1561/112.00000441

Dale, V. H., Tharp, M. L., Lannom, K. O., & Hodges, D. G. (2010). Modeling transient response of forests to climate change. *Science of the Total Environment*, *408*(8). https://doi.org/10.1016/j.scitotenv.2009.11.050

Dellink, R., Chateau, J., Lanzi, E., & Magné, B. (2017). Long-term economic growth projections in the Shared Socioeconomic Pathways. *Global Environmental Change*, *42*. https://doi.org/10.1016/j.gloenvcha.2015.06.004

Delzeit, R., Heimann, T., Schuenemann, F., Söder, M., Zabel, F., & Hosseini, M. (2021). Scenarios for an impact assessment of global bioeconomy strategies: Results from a co-design process. *Research in Globalization*, *3*. https://doi.org/10.1016/j.resglo.2021.100060

Dias, M. A. de P., Vianna, J. N. de S., & Felby, C. (2016). Sustainability in the prospective scenarios methods: A case study of scenarios for biodiesel industry in Brazil, for 2030. *Futures*, *82*. https://doi.org/10.1016/j.futures.2016.06.005

Ding, H., Chiabai, A., Silvestri, S., & Nunes, P. A. L. D. (2016). Valuing climate change impacts on European forest ecosystems. *Ecosystem Services*, *18*. https://doi.org/10.1016/j.ecoser.2016.02.039

Ding, H., Silvestri, S., Chiabai, A., & Nunes, P. A. L. D. (2012). A Hybrid Approach to the Valuation of Climate Change Effects on Ecosystem Services: Evidence from the European Forests. *SSRN Electronic Journal*. https://doi.org/10.2139/ssrn.1603826

Drake, H. F., Rivest, R. L., Edelman, A., & Deutch, J. (2021). A simple model for assessing climate control trade-offs and responding to unanticipated climate outcomes. *Environmental Research Letters*, *16*(10). https://doi.org/10.1088/1748-9326/ac243e

Eggers, J., Lindner, M., Zudin, S., Zaehle, S., & Liski, J. (2008). Impact of changing wood demand, climate and land use on European forest resources and carbon stocks during the 21st century. *Global Change Biology*, *14*(10). https://doi.org/10.1111/j.1365-2486.2008.01653.x

Engström, K., Olin, S., Rounsevell, M. D. A., Brogaard, S., van Vuuren, D. P., Alexander, P., Murray-Rust, D., & Arneth, A. (2016). Assessing uncertainties in global cropland futures using a conditional probabilistic modelling framework. *Earth System Dynamics*, *7*(4). https://doi.org/10.5194/esd-7-893-2016

Fenhann, J. (2000). Industrial non-energy, non-CO2 greenhouse gas emissions. *Technological Forecasting and Social Change*, *63*(2–3). https://doi.org/10.1016/S0040-1625(99)00107-9

Freund, E. R., Abbaspour, K. C., & Lehmann, A. (2017). Water resources of the Black Sea Catchment under future climate and landuse change projections. *Water (Switzerland)*, *9*(8). https://doi.org/10.3390/w9080598

Fronzek, S., Carter, T. R., & Jylhä, K. (2012). Representing two centuries of past and future climate for assessing risks to biodiversity in Europe. *Global Ecology and Biogeography*, *21*(1). https://doi.org/10.1111/j.1466-8238.2011.00695.x

Garzón, M. B., Sánchez De Dios, R., & Sainz Ollero, H. (2008). The evolution of the Pinus sylvestris L. area in the Iberian Peninsula from the last glacial maximum to 2100 under climate change. *Holocene*, *18*(5). https://doi.org/10.1177/0959683608091781

Gerst, M. D., Wang, P., & Borsuk, M. E. (2013). Discovering plausible energy and economic futures under global change using multidimensional scenario discovery. *Environmental Modelling and Software*, *44*. https://doi.org/10.1016/j.envsoft.2012.09.001

Goklany, I. M. (2005). Evidence to the House of Lords Select Committee on Economic Affairs on aspects of the economics of climate change. *Energy and Environment*, *16*(3–4). https://doi.org/10.1260/0958305054672312

Graux, A. I., Lardy, R., Bellocchi, G., & Soussana, J. F. (2012). Global warming potential of French grassland-based dairy livestock systems under climate change. *Regional Environmental Change*, *12*(4). https://doi.org/10.1007/s10113-012-0289-2

Gudmundsson, S. V., & Anger, A. (2012). Global carbon dioxide emissions scenarios for aviation derived from IPCC storylines: A meta-analysis. *Transportation Research Part D: Transport and Environment*, *17*(1). https://doi.org/10.1016/j.trd.2011.09.010

Hainsch, K., Löffler, K., Burandt, T., Auer, H., Crespo del Granado, P., Pisciella, P., & Zwickl-Bernhard, S. (2022). Energy transition scenarios: What policies, societal attitudes, and technology developments will realize the EU Green Deal? *Energy*, *239*. https://doi.org/10.1016/j.energy.2021.122067

Hazeleger, W., Van Den Hurk, B. J. J. M., Min, E., Van Oldenborgh, G. J., Petersen, A. C., Stainforth, D. A., Vasileiadou, E., & Smith, L. A. (2015). Tales of future weather. *Nature Climate Change*, *5*(2), 107–113. https://doi.org/10.1038/nclimate2450

Henriques, C. (2007). Hydrology and water resources management in East Anglia and North West England in the context of climate and socio-economic change. *Applied Sciences*.

Hofstra, N., & Vermeulen, L. C. (2016). Impacts of population growth, urbanisation and sanitation changes on global human Cryptosporidium emissions to surface water. *International Journal of Hygiene and Environmental Health*, *219*(7). https://doi.org/10.1016/j.ijheh.2016.06.005

Holmgren, S., Pever, M., & Fischer, K. (2019). Constructing low-carbon futures? Competing storylines in the Estonian energy sector's translation of EU energy goals. *Energy Policy*, *135*. https://doi.org/10.1016/j.enpol.2019.111063

Honjo, K., Gomi, K., Kanamori, Y., Takahashi, K., & Matsuhashi, K. (2021). Long-term projections of economic growth in the 47 prefectures of Japan: An application of Japan shared socioeconomic pathways. *Heliyon*, *7*(3). https://doi.org/10.1016/j.heliyon.2021.e06412

Hurri, K. (2020). Rethinking climate leadership: Annex I countries' expectations for China's leadership role in the post-Paris UN climate negotiations. *Environmental Development*, *35*. https://doi.org/10.1016/j.envdev.2020.100544

Hutchins, M. G., Abesser, C., Prudhomme, C., Elliott, J. A., Bloomfield, J. P., Mansour, M. M., & Hitt, O. E. (2018). Combined impacts of future land-use and climate stressors on water resources and quality in groundwater and surface waterbodies of the upper Thames river basin, UK. *Science of the Total Environment*, *631–632*. https://doi.org/10.1016/j.scitotenv.2018.03.052

Immerzeel, W. (2008). Historical trends and future predictions of climate variability in the Brahmaputra basin. *International Journal of Climatology*, *28*(2). https://doi.org/10.1002/joc.1528

Jalilzadehazhari, E., Vadiee, A., & Johansson, J. (2021). Subsidies required for installing renewable energy supply systems considering variations in future climate conditions. *Journal of Building Engineering*, *35*. https://doi.org/10.1016/j.jobe.2020.101999

Jiang, K., Masui, T., Morita, T., & Matsuoka, Y. (2000). Long-term GHG emission scenarios for Asia-Pacific and the world. *Technological Forecasting and Social Change*, *63*(2–3). https://doi.org/10.1016/S0040-1625(99)00110-9

Jiang, K., Morita, T., Masui, T., & Matsuoka, Y. (2000). Global long-term greenhouse gas mitigation emission scenarios based on AIM. *Environmental Economics and Policy Studies*, *3*(2). https://doi.org/10.1007/BF03354039

Jorda-Capdevila, D., Gampe, D., Huber García, V., Ludwig, R., Sabater, S., Vergoñós, L., & Acuña, V. (2019). Impact and mitigation of global change on freshwater-related ecosystem services in Southern Europe. *Science of the Total Environment*, *651*. https://doi.org/10.1016/j.scitotenv.2018.09.228

Kangas, J. (2019). Picturing two modernities: Ecological modernisation and the media imagery of climate change. *Nordicom Review*, *40*(1). https://doi.org/10.2478/nor-2019-0003.

Karhunmaa, K. (2016). Opening up storylines of co-benefits in voluntary carbon markets: An analysis of household energy technology projects in developing countries. *Energy Research and Social Science*, *14*. https://doi.org/10.1016/j.erss.2016.01.011

Kim, J., Choi, J., Choi, C., & Park, S. (2013). Impacts of changes in climate and land use/land cover under IPCC RCP scenarios on streamflow in the Hoeya River Basin, Korea. *Science of the Total Environment*, *452–453*. https://doi.org/10.1016/j.scitotenv.2013.02.005

Knevels, R., Petschko, H., Proske, H., Leopold, P., Maraun, D., & Brenning, A. (2020). Event-based landslide modeling in the styrian basin, Austria: Accounting for time-varying rainfall and land cover. *Geosciences (Switzerland)*, *10*(6). https://doi.org/10.3390/geosciences10060217

Kros, J., Bakker, M. M., Reidsma, P., Kanellopoulos, A., Jamal Alam, S., & de Vries, W. (2015). Impacts of agricultural changes in response to climate and socioeconomic change on nitrogen deposition in nature reserves. *Landscape Ecology*, *30*(5). https://doi.org/10.1007/s10980-014-0131-y

Leimbach, M., Labriet, M., Bonsch, M., Dietrich, J. P., Kanudia, A., Mouratiadou, I., Popp, A., & Klein, D. (2016). Robust strategies of climate change mitigation in interacting energy, economy and land use systems. *International Journal of Climate Change Strategies and Management*, *8*(5). https://doi.org/10.1108/IJCCSM-09-2015-0135

Leipold, S. (2021). Transforming ecological modernization 'from within' or perpetuating it? The circular economy as EU environmental policy narrative. *Environmental Politics*, *30*(6), 1045–1067. https://doi.org/10.1080/09644016.2020.1868863

Leung, A., Burke, M., Perl, A., & Cui, J. (2018). The peak oil and oil vulnerability discourse in urban transport policy: A comparative discourse analysis of Hong Kong and Brisbane. *Transport Policy*, *65*. https://doi.org/10.1016/j.tranpol.2017.03.023

Lin, W., Sun, Y., Nijhuis, S., & Wang, Z. (2020). Scenario-based flood risk assessment for urbanizing deltas using future land-use simulation (FLUS): Guangzhou Metropolitan Area as a case study. *Science of the Total Environment*, *739*. https://doi.org/10.1016/j.scitotenv.2020.139899

Lovell, H., Bulkeley, H., & Owens, S. (2009). Converging agendas? Energy and climate change policies in the UK. *Environment and Planning C: Government and Policy*, *27*(1). https://doi.org/10.1068/c0797j

Lucas, C., & Warman, R. (2018). Disrupting polarized discourses: Can we get out of the ruts of environmental conflicts? *Environment and Planning C: Politics and Space*, *36*(6). https://doi.org/10.1177/2399654418772843

Mack, L., Andersen, H. E., Beklioğlu, M., Bucak, T., Couture, R. M., Cremona, F., Ferreira, M. T., Hutchins, M. G., Mischke, U., Molina-Navarro, E., Rankinen, K., Venohr, M., & Birk, S. (2019). The future depends on what we do today – Projecting Europe's surface water quality into three different future scenarios. *Science of the Total Environment*, *668*. https://doi.org/10.1016/j.scitotenv.2019.02.251

Mancosu, E., Gago-Silva, A., Barbosa, A., de Bono, A., Ivanov, E., Lehmann, A., & Fons, J. (2015). Future land-use change scenarios for the Black Sea catchment. *Environmental Science and Policy*, *46*. https://doi.org/10.1016/j.envsci.2014.02.008

Markard, J., Rinscheid, A., & Widdel, L. (2021). Analyzing transitions through the lens of discourse networks: Coal phase-out in Germany. *Environmental Innovation and Societal Transitions*, *40*. https://doi.org/10.1016/j.eist.2021.08.001

Mather-Gratton, Z. J., Larsen, S., & Bentsen, N. S. (2021). Understanding the sustainability debate on forest biomass for energy in Europe: A discourse analysis. *PLoS ONE*, *16*(2 February). https://doi.org/10.1371/journal.pone.0246873

Matthews, T. (2015). Storylines of institutional responses to climate change as a transformative stressor: the case of regional planning in South East Queensland, Australia. *Environment and Planning C: Government and Policy*, *33*(5). https://doi.org/10.1177/0263774X15610054

Matthews, T., & Marston, G. (2019). How environmental storylines shaped regional planning policies in South East Queensland, Australia: A long-term analysis. *Land Use Policy*, *85*. https://doi.org/10.1016/j.landusepol.2019.03.042

Mayrhofer, J., & Gupta, J. (2016). The politics of co-benefits in India's energy sector. *Environment and Planning C: Government and Policy*, *34*(7). https://doi.org/10.1177/0263774X15619629

Melo, I., Turnhout, E., & Arts, B. (2014). Integrating multiple benefits in market-based climate mitigation schemes: The case of the Climate, Community and Biodiversity certification scheme. *Environmental Science and Policy*, *35*. https://doi.org/10.1016/j.envsci.2013.02.010

Melsen, L. A., Addor, N., Mizukami, N., Newman, A. J., Torfs, P. J. J. F., Clark, M. P., Uijlenhoet, R., & Teuling, A. J. (2018). Mapping (dis)agreement in hydrologic projections. *Hydrology and Earth System Sciences*, *22*(3). https://doi.org/10.5194/hess-22-1775-2018

Merino, G., Barange, M., & Mullon, C. (2010). Climate variability and change scenarios for a marine commodity: Modelling small pelagic fish, fisheries and fishmeal in a globalized market. *Journal of Marine Systems*, *81*(1–2). https://doi.org/10.1016/j.jmarsys.2009.12.010

Mirasgedis, S., Sarafidis, Y., Georgopoulou, E., Kotroni, V., Lagouvardos, K., & Lalas, D. P. (2007). Modeling framework for estimating impacts of climate change on electricity demand at regional level: Case of Greece. *Energy Conversion and Management*, *48*(5). https://doi.org/10.1016/j.enconman.2006.10.022

Molina-Navarro, E., Andersen, H. E., Nielsen, A., Thodsen, H., & Trolle, D. (2018). Quantifying the combined effects of land use and climate changes on stream flow and nutrient loads: A modelling approach in the Odense Fjord catchment (Denmark). *Science of the Total Environment*, *621*. https://doi.org/10.1016/j.scitotenv.2017.11.251

Mori, S. (2000a). Effects of carbon emission mitigation options under carbon concentration stabilization scenarios. *Environmental Economics and Policy Studies*, *3*(2). https://doi.org/10.1007/BF03354033

Mori, S. (2000b). The development of greenhouse gas emissions scenarios using an extension of the MARIA model for the assessment of resource and energy technologies. *Technological Forecasting and Social Change*, *63*(2–3). https://doi.org/10.1016/S0040-1625(99)00102-X

Morin, X., & Chuine, I. (2014). Will tree species experience increased frost damage due to climate change because of changes in leaf phenology? *Canadian Journal of Forest Research*, *44*(12). https://doi.org/10.1139/cjfr-2014-0282

Morin, X., Viner, D., & Chuine, I. (2008). Tree species range shifts at a continental scale: New predictive insights from a process-based model. *Journal of Ecology*, *96*(4). https://doi.org/10.1111/j.1365-2745.2008.01369.x

Morton, T. (2021). Contesting Coal, Contesting Climate: Materializing the Social Drama of Climate Change in Australia and Germany. *Environmental Communication*, *15*(4). https://doi.org/10.1080/17524032.2020.1865428

Mullens, E. D., & McPherson, R. A. (2019). Quantitative scenarios for future hydrologic extremes in the U.S. Southern Great Plains. *International Journal of Climatology*, *39*(5). https://doi.org/10.1002/joc.5979

Müller, C., Eickhout, B., Zaehle, S., Bondeau, A., Cramer, W., & Lucht, W. (2007). Effects of changes in CO2, climate, and land use on the carbon balance of the land biosphere during the 21st century. *Journal of Geophysical Research: Biogeosciences*, *112*(2). https://doi.org/10.1029/2006JG000388

Nauels, A., Rogelj, J., Schleussner, C. F., Meinshausen, M., & Mengel, M. (2017). Linking sea level rise and socioeconomic indicators under the Shared Socioeconomic Pathways. *Environmental Research Letters*, *12*(11). https://doi.org/10.1088/1748-9326/aa92b6

Nechifor, V., & Winning, M. (2019). Global crop output and irrigation water requirements under a changing climate. *Heliyon*, *5*(3). https://doi.org/10.1016/j.heliyon.2019.e01266

Neubauer, R., & Gunster, S. (2019). Enemies at the Gateway: Regional Populist Discourse and the Fight against Oil Pipelines on Canada's West Coast. *Frontiers in Communication*, *4*. https://doi.org/10.3389/fcomm.2019.00061

Newing, H. S. (2009). Traditional knowledge in international forest policy: contested meanings and divergent discourses. *Journal of Integrative Environmental Sciences*, *6*(3). https://doi.org/10.1080/19438150903090491

Nicholls, R. J. (2004). Coastal flooding and wetland loss in the 21st century: Changes under the SRES climate and socio-economic scenarios. *Global Environmental Change*, *14*(1). https://doi.org/10.1016/j.gloenvcha.2003.10.007

Nielsen, T. D. (2016). From REDD+ forests to green landscapes? Analyzing the emerging integrated landscape approach discourse in the UNFCCC. *Forest Policy and Economics*, *73*. https://doi.org/10.1016/j.forpol.2016.09.006

Nunes, J. P., Jacinto, R., & Keizer, J. J. (2017). Combined impacts of climate and socio-economic scenarios on irrigation water availability for a dry Mediterranean reservoir. *Science of the Total Environment*, *584–585*. https://doi.org/10.1016/j.scitotenv.2017.01.131

Nussbaumer, S., Schaub, Y., Huggel, C., & Walz, A. (2014). Risk estimation for future glacier lake outburst floods based on local land-use changes. *Natural Hazards and Earth System Sciences*, *14*(6). https://doi.org/10.5194/nhess-14-1611-2014

Obersteiner, M., Alexandrov, G., Benítez, P. C., McCallum, I., Kraxner, F., Riahi, K., Rokityanskiy, D., & Yamagata, Y. (2006). Global supply of biomass for energy and carbon sequestration from

afforestation/reforestation activities. *Mitigation and Adaptation Strategies for Global Change*, *11*(5–6). https://doi.org/10.1007/s11027-006-9031-z

Pais, S., Aquilué, N., Campos, J., Sil, Â., Marcos, B., Martínez-Freiría, F., Domínguez, J., Brotons, L., Honrado, J. P., & Regos, A. (2020). Mountain farmland protection and fire-smart management jointly reduce fire hazard and enhance biodiversity and carbon sequestration. *Ecosystem Services*, *44*. https://doi.org/10.1016/j.ecoser.2020.101143

Parish, E. S., Kodra, E., Steinhaeuser, K., & Ganguly, A. R. (2012). Estimating future global per capita water availability based on changes in climate and population. *Computers and Geosciences*, *42*. https://doi.org/10.1016/j.cageo.2012.01.019

Pauliuk, S., Fishman, T., Heeren, N., Berrill, P., Tu, Q., Wolfram, P., & Hertwich, E. G. (2021). Linking service provision to material cycles: A new framework for studying the resource efficiency–climate change (RECC) nexus. *Journal of Industrial Ecology*, *25*(2). https://doi.org/10.1111/jiec.13023

Politti, E., Egger, G., Angermann, K., Rivaes, R., Blamauer, B., Klösch, M., Tritthart, M., & Habersack, H. (2014). Evaluating climate change impacts on Alpine floodplain vegetation. *Hydrobiologia*, *737*(1). https://doi.org/10.1007/s10750-013-1801-5

Popp, A., Calvin, K., Fujimori, S., Havlik, P., Humpenöder, F., Stehfest, E., Bodirsky, B. L., Dietrich, J. P., Doelmann, J. C., Gusti, M., Hasegawa, T., Kyle, P., Obersteiner, M., Tabeau, A., Takahashi, K., Valin, H., Waldhoff, S., Weindl, I., Wise, M., … Vuuren, D. P. van. (2017). Land-use futures in the shared socio-economic pathways. *Global Environmental Change*, *42*. https://doi.org/10.1016/j.gloenvcha.2016.10.002

Poulter, B., Aragão, L., Heyder, U., Gumpenberger, M., Heinke, J., Langerwisch, F., Rammig, A., Thonicke, K., & Cramer, W. (2010). Net biome production of the Amazon Basin in the 21st century. *Global Change Biology*, *16*(7). https://doi.org/10.1111/j.1365-2486.2009.02064.x

Poulter, B., Hattermann, F., Hawkins, E., Zaehle, S., Sitch, S., Restrepo-Coupe, N., Heyder, U., & Cramer, W. (2010). Robust dynamics of Amazon dieback to climate change with perturbed ecosystem model parameters. *Global Change Biology*, *16*(9). https://doi.org/10.1111/j.1365-2486.2009.02157.x

Rankinen, K., Cano Bernal, J. E., Holmberg, M., Vuorio, K., & Granlund, K. (2019). Identifying multiple stressors that influence eutrophication in a Finnish agricultural river. *Science of the Total Environment*, *658*. https://doi.org/10.1016/j.scitotenv.2018.12.294

Rankinen, K., Peltonen-Sainio, P., Granlund, K., Ojanen, H., Laapas, M., Hakala, K., Sippel, K., Helenius, J., & Forsius, M. (2013). Climate change adaptation in arable land use, and impact on nitrogen load at catchment scale in northern agriculture. *Agricultural and Food Science*, *22*(3). https://doi.org/10.23986/afsci.7500

Riahi, K., & Roehrl, R. A. (2000). Greenhouse gas emissions in a dynamics-as-usual scenario of economic and energy development. *Technological Forecasting and Social Change*, *63*(2–3). https://doi.org/10.1016/S0040-1625(99)00111-0

Rogge, K. S., Pfluger, B., & Geels, F. W. (2020). Transformative policy mixes in socio-technical scenarios: The case of the low-carbon transition of the German electricity system (2010–2050). *Technological Forecasting and Social Change*, *151*. https://doi.org/10.1016/j.techfore.2018.04.002

Rogner, H. H., McDonald, A., & Riahi, K. (2008). Long-term performance targets for nuclear energy. Part 1: The global scenario context. *International Journal of Global Energy Issues*, *30*(1–4). https://doi.org/10.1504/ijgei.2008.019856

Rounsevell, M. D. A., Ewert, F., Reginster, I., Leemans, R., & Carter, T. R. (2005). Future scenarios of European agricultural land use: II. Projecting changes in cropland and grassland. *Agriculture, Ecosystems and Environment*, *107*(2–3). https://doi.org/10.1016/j.agee.2004.12.002

Rounsevell, M. D. A., & Metzger, M. J. (2010). Developing qualitative scenario storylines for environmental change assessment. In *Wiley Interdisciplinary Reviews: Climate Change* (Vol. 1, Issue 4, pp. 606–619). Wiley-Blackwell. https://doi.org/10.1002/wcc.63

Samson, E. A., Boykin, K. G., Kepner, W. G., Andersen, M. C., & Fernald, A. (2018). Evaluating biodiversity metric response to forecasted land use change in the Northern Rio Grande Basin. *Environments - MDPI*, *5*(8). https://doi.org/10.3390/environments5080091

Samsuri, R., Kamal, F., & Hayder, G. (2018). Climate Change Impact Assessment to the proposed coal fired power plant project at East Coast of Peninsular Malaysia. *International Journal of Engineering and Technology(UAE)*, *7*(4). https://doi.org/10.14419/ijet.v7i4.35.22906

Sankovski, A., Barbour, W., & Pepper, W. (2000). Quantification of the IS99 emission scenario storylines using the atmospheric stabilization framework. *Technological Forecasting and Social Change*, *63*(2–3). https://doi.org/10.1016/S0040-1625(99)00100-6

Schaldach, R., Göpel, J., & Klingler, M. (2017). The role of future land-use change in Southern Amazonia to reach the aims of Brazil's national climate plan. *Erdkunde*, *71*(3). https://doi.org/10.3112/erdkunde.2017.03.04

Schenkel, W. (2000). From clean air to climate policy in the netherlands and switzerland: How two small states deal with a global problem. *Swiss Political Science Review*, *6*(1). https://doi.org/10.1002/j.1662-6370.2000.tb00289.x

Sellers, S., & Ebi, K. L. (2018). Climate change and health under the shared socioeconomic pathway framework. *International Journal of Environmental Research and Public Health*, *15*(1). https://doi.org/10.3390/ijerph15010003

Semadeni-Davies, A., Hernebring, C., Svensson, G., & Gustafsson, L. G. (2008). The impacts of climate change and urbanisation on drainage in Helsingborg, Sweden: Suburban stormwater. *Journal of Hydrology*, *350*(1–2). https://doi.org/10.1016/j.jhydrol.2007.11.006

Serpa, D., Nunes, J. P., Keizer, J. J., & Abrantes, N. (2017). Impacts of climate and land use changes on the water quality of a small Mediterranean catchment with intensive viticulture. *Environmental Pollution*, *224*. https://doi.org/10.1016/j.envpol.2017.02.026

Serpa, D., Nunes, J. P., Santos, J., Sampaio, E., Jacinto, R., Veiga, S., Lima, J. C., Moreira, M., Corte-Real, J., Keizer, J. J., & Abrantes, N. (2015). Impacts of climate and land use changes on the hydrological and erosion processes of two contrasting Mediterranean catchments. *Science of the Total Environment*, *538*. https://doi.org/10.1016/j.scitotenv.2015.08.033

Shepherd, T. G. (2016). A Common Framework for Approaches to Extreme Event Attribution. In *Current Climate Change Reports* (Vol. 2, Issue 1, pp. 28–38). Springer. https://doi.org/10.1007/s40641-016-0033-y

Shepherd, T. G., Boyd, E., Calel, R. A., Chapman, S. C., Dessai, S., Dima-West, I. M., Fowler, H. J., James, R., Maraun, D., Martius, O., Senior, C. A., Sobel, A. H., Stainforth, D. A., Tett, S. F. B., Trenberth, K. E., van den Hurk, B. J. J. M., Watkins, N. W., Wilby, R. L., & Zenghelis, D. A. (2018). Storylines: an alternative approach to representing uncertainty in physical aspects of climate change. *Climatic Change*, *151*(3–4), 555–571. https://doi.org/10.1007/s10584-018-2317-9

Sheshukov, A. Y., Siebenmorgen, C. B., & Douglas-Mankin, K. R. (2011). Seasonal and annual impacts of climate change on watershed response using an ensemble of global climate models. *Transactions of the ASABE*, *54*(6).

Shindell, D. T., Levy, H., Schwarzkopf, M. D., Horowitz, L. W., Lamarque, J. F., & Faluvegi, G. (2008). Multimodel projections of climate change from short-lived emissions due to human activities. *Journal of Geophysical Research Atmospheres*, *113*(11). https://doi.org/10.1029/2007JD009152

Sjøeng, A. M. S., Kaste, Ø., & Wright, R. F. (2009). Modelling future no3 leaching from an upland headwater catchment in sw norway using the magic model: Ii. Simulation of future nitrate leaching given scenarios of climate change and nitrogen deposition. *Hydrology Research*, *40*(2–3). https://doi.org/10.2166/nh.2009.068

Spencer, T., & Dubash, N. K. (2022). Scenarios for different 'Future Indias': sharpening energy and climate modelling tools. *Climate Policy*, *22*(1). https://doi.org/10.1080/14693062.2021.1973361

Strasser, U., Förster, K., Formayer, H., Hofmeister, F., Marke, T., Meißl, G., Nadeem, I., Stotten, R., & Schermer, M. (2019). Storylines of combined future land use and climate scenarios and their hydrological impacts in an Alpine catchment (Brixental/Austria). *Science of the Total Environment*, *657*. https://doi.org/10.1016/j.scitotenv.2018.12.077

Taylor, B., Wallington, T., Heyenga, S., & Harman, B. (2014). Urban Growth and Climate Adaptation in Australia: Divergent Discourses and Implications for Policy-making. *Urban Studies*, *51*(1). https://doi.org/10.1177/0042098013484529

Teräväinen, T. (2010). Political opportunities and storylines in finnish climate policy negotiations. *Environmental Politics*, *19*(2). https://doi.org/10.1080/09644010903574475

Thuiller, W., Broennimann, O., Hughes, G., Alkemade, J. R. M., Midgley, G. F., & Corsi, F. (2006). Vulnerability of African mammals to anthropogenic climate change under conservative land transformation assumptions. *Global Change Biology*, *12*(3). https://doi.org/10.1111/j.1365-2486.2006.01115.x

Thuiller, W., Lavorel, S., Araújo, M. B., Sykes, M. T., & Prentice, I. C. (2005). Climate change threats to plant diversity in Europe. *Proceedings of the National Academy of Sciences of the United States of America*, *102*(23). https://doi.org/10.1073/pnas.0409902102

Toke, D. (2013). Climate change and the nuclear securitisation of UK energy policy. *Environmental Politics*, *22*(4). https://doi.org/10.1080/09644016.2013.806630

Tripathy, R., Ray, S. S., Kaur, H., Jalota, S. K., Bal, S. K., & Panigrahy, S. (2012). UNDERSTANDING SPATIAL VARIABILITY OF CROPPING SYSTEM RESPONSE TO CLIMATE CHANGE IN PUNJAB STATE OF INDIA USING REMOTE SENSING DATA AND SIMULATION MODEL. *The International Archives of the Photogrammetry, Remote Sensing and Spatial Information Sciences*, *XXXVIII-8/W20*. https://doi.org/10.5194/isprsarchives-xxxviii-8-w20-29-2011

van Eck, C. W., & Feindt, P. H. (2021). Parallel routes from Copenhagen to Paris: climate discourse in climate sceptic and climate activist blogs. *Journal of Environmental Policy and Planning*. https://doi.org/10.1080/1523908X.2021.2000376

van Vuuren, D. P., Stehfest, E., Gernaat, D. E. H. J., Doelman, J. C., van den Berg, M., Harmsen, M., de Boer, H. S., Bouwman, L. F., Daioglou, V., Edelenbosch, O. Y., Girod, B., Kram, T., Lassaletta, L., Lucas, P. L., van Meijl, H., Müller, C., van Ruijven, B. J., van der Sluis, S., & Tabeau, A. (2017). Energy, land-use and greenhouse gas emissions trajectories under a green growth paradigm. *Global Environmental Change*, *42*. https://doi.org/10.1016/j.gloenvcha.2016.05.008

Vogel, S. M., Monsarrat, S., Pasgaard, M., Buitenwerf, R., Li, W., Gordon, C. E., Jarvie, S., Mata, J., Pearce, E. A. ., & Svenning, J.-C. (2021). A multi-disciplinary perspective to nuance the narrative of tree planting as a nature-based solution. *SocArXiv*.

Wang, P., Gerst, M. D., & Borsuk, M. E. (2013). Exploring energy and economic futures using agent-based modeling and scenario discovery. *Understanding Complex Systems*. https://doi.org/10.1007/978-1-4614-8606-0_13

Wheeler, S. M., Tomuta, M., Haden, V. R., & Jackson, L. E. (2013). The impacts of alternative patterns of urbanization on greenhouse gas emissions in an agricultural county. *Journal of Urbanism*, *6*(3). https://doi.org/10.1080/17549175.2013.777356

Yuan, F., Xie, Z. H., Liu, Q., & Xia, J. (2005). Simulating hydrologic changes with climate change scenarios in the Haihe River Basin. *Pedosphere*, *15*(5).

Zaehle, S., Bondeau, A., Carter, T. R., Cramer, W., Erhard, M., Prentice, I. C., Reginster, I., Rounsevell, M. D. A., Sitch, S., Smith, B., Smith, P. C., & Sykes, M. (2007). Projected changes in terrestrial carbon storage in Europe under climate and land-use change, 1990-2100. *Ecosystems*, *10*(3). https://doi.org/10.1007/s10021-007-9028-9

Zelli, F., Nielsen, T. D., & Dubber, W. (2019). Seeing the forest for the trees: Identifying discursive convergence and dominance in complex redd+ governance. *Ecology and Society*, *24*(1). https://doi.org/10.5751/ES-10632-240110
